# How index selection, compression, and recording schedule impact the description of ecological soundscapes

**DOI:** 10.1002/ece3.8042

**Published:** 2021-08-26

**Authors:** Becky E. Heath, Sarab S. Sethi, C. David L. Orme, Robert M. Ewers, Lorenzo Picinali

**Affiliations:** ^1^ Dyson School of Design Engineering Imperial College London London UK; ^2^ Department of Life Sciences Imperial College London London UK; ^3^ Department of Mathematics Imperial College London London UK

## Abstract

1. Acoustic indices derived from environmental soundscape recordings are being used to monitor ecosystem health and vocal animal biodiversity. Soundscape data can quickly become very expensive and difficult to manage, so data compression or temporal down‐sampling are sometimes employed to reduce data storage and transmission costs. These parameters vary widely between experiments, with the consequences of this variation remaining mostly unknown.
2. We analyse field recordings from North‐Eastern Borneo across a gradient of historical land use. We quantify the impact of experimental parameters (MP3 compression, recording length and temporal subsetting) on soundscape descriptors (Analytical Indices and a convolutional neural net derived AudioSet Fingerprint). Both descriptor types were tested for their robustness to parameter alteration and their usability in a soundscape classification task.
3. We find that compression and recording length both drive considerable variation in calculated index values. However, we find that the effects of this variation and temporal subsetting on the performance of classification models is minor: performance is much more strongly determined by acoustic index choice, with Audioset fingerprinting offering substantially greater (12%–16%) levels of classifier accuracy, precision and recall.
4. We advise using the AudioSet Fingerprint in soundscape analysis, finding superior and consistent performance even on small pools of data. If data storage is a bottleneck to a study, we recommend Variable Bit Rate encoded compression (quality = 0) to reduce file size to 23% file size without affecting most Analytical Index values. The AudioSet Fingerprint can be compressed further to a Constant Bit Rate encoding of 64 kb/s (8% file size) without any detectable effect. These recommendations allow the efficient use of restricted data storage whilst permitting comparability of results between different studies.

## Graphical abstract

Soundscapes were recorded from different forest structures in Malaysian Borneo. Data collection variation was simulated, and all data groups were analyzed via usual acoustic indices and a CNN‐derived AudioSet Fingerprint. The effect of variation in data collection was compared between the two types of soundscape descriptor, finding the AudioSet Fingerprint to be a stronger and more robust descriptor of soundscapes.

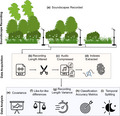

## 1 INTRODUCTION

Animal vocalizations come together with abiotic and human‐made sounds to form soundscapes. These soundscapes can be recorded and quantified across large temporal and spatial dimensions to monitor species populations or infer community‐level metrics such as biodiversity (Eldridge et al., 2018; Gómez et al., 2018; Roca & Proulx, 2016). Monitoring is crucial to effectively respond to threats such as disease, species loss, and overlogging (Rapport, 1989; Rapport et al., 1998). Previously, the use of in situ expert listeners to monitor species presence and abundance was common (Huff et al., 2000) but is costly and time‐consuming; can damage habitats; and is prone to narrow focus and observer bias (Costello et al., 2016; Fitzpatrick et al., 2009). Advances in portable computing now permit remote recording of soundscapes, but produce a volume of data that is very time‐consuming to review manually, leading to the development of automated, or semiautomated, methods of analysis (Sethi, Jones, et al., 2020; Towsey et al., 2016).

Soundscape composition is primarily assessed using acoustic indices which describe the soundscape in an abstracted form. Analytical Indices are a type of acoustic index which are summary statistics that describe the distribution of acoustic energy within the recording (Towsey et al., 2014)—over 60 of which have been designed to capture aspects of biodiversity (Buxton et al., 2018; Sueur et al., 2014). These are commonly used in combination to compare the occupancy of acoustic niches, temporal variation, and the general level of acoustic activity (Bradfer‐Lawrence et al., 2019) across ecological gradients or in classification tasks (Gómez et al., 2018). These approaches have provided novel insight into ecosystems across the world (Buxton et al., 2018; Eldridge et al., 2018; Fuller et al., 2015; Sueur et al., 2019) but are not foolproof and often have poor transferability (Bohnenstiehl et al., 2018; Mammides et al., 2017). This may result from a lack of standardization: differing index selection, data storage methods, and recording protocols, which all lead to unassessed variation in experimental outputs (Araya‐Salas et al., 2019; Bradfer‐Lawrence et al., 2019; Sugai et al., 2019).

The output vector from the AudioSet convolutional neural net (CNN; Gemmeke et al., 2017; Hershey et al., 2017) is an attractive replacement for Analytical Indices. This pretrained, general‐purpose audio classification algorithm generates a multidimensional acoustic fingerprint of a soundscape which can be used as a more effective suite of acoustic indices (Sethi, Jones, et al., 2020). The AudioSet CNN is trained on two million human‐labeled anthropogenic and environmental audio samples, potentially giving it both greater transferability and discrimination than typical ecoacoustic training datasets. Unlike Analytical Indices, however, extra analysis (such as training classifiers/predictive models) is necessary to relate the AudioSet Fingerprint to ecological processes and states.

In ecoacoustics, a continuous uncompressed or lossless recording is generally recommended (Browning et al., 2017; Villanueva‐Rivera et al., 2011), but generates huge files. We considered two commonly used approaches to reducing storage requirements (Towsey, 2018). Firstly, MP3 compression, which is widely used in ecoacoustic studies (e.g., Saito et al., 2015; Sethi, Jones, et al., 2018; Zhang et al., 2016): This lossy encoding removes acoustic information inaudible to *human* listeners (Sterne, 2012) but is suspected of removing ecologically important data (e.g., Sugai et al., 2019; Towsey et al., 2016). Araya‐Salas et al. (2019) have recently shown that ecological information is lost under high compression from recordings of isolated animal calls; however, it is not known if this extends to recordings of noisier whole soundscapes.

Secondly, recording schedules also vary in ecoacoustic studies (Sugai et al., 2019). Bradfer‐Lawrence et al. (2019) showed that longer and more continuous schedules give more stable Analytical Index values. However, ecoacoustic composition varies with time of day (Bradfer‐Lawrence et al., 2019; Fuller et al., 2015; Sethi, Jones, et al., 2020) and so reducing recording periods with temporal subsetting may reduce temporal variation and improve classification (Sugai et al., 2019) even with reduced data. Similarly, index calculation on longer recordings may average away anomalous calls and short‐term patterns.

While clear standards are crucial for collaborative research in ecoacoustics, there is uncertainty in the literature on the impacts of the selection of index type, compression level, and recording schedule on the quantification and classification of ecological soundscapes. Here, we:

1. investigated the impact of index selection on the accuracy of a random forest classifier;
2. described the effects of compression, recording length, and temporal subsetting on the values, variance, and classification performance of indices.

In describing how well ecological information is stored in acoustic data under different recording decisions, we identified stronger standards to improve classifier accuracy, precision, and recall and provided a basis for comparison among studies.

## 2 METHODS AND MATERIALS

### 2.1 Study area

Acoustic samples were collected in Sabah, Malaysian Borneo, at the Stability of Altered Forest Ecosystems (SAFE) project: a large‐scale ecological experiment on habitat loss and fragmentation effects on tropical forests (Ewers et al., 2011) which included sites in the Kalabakan Forest Reserve (KFR). Historically, logging within KFR has been heterogeneous, reflecting habitat modifications in the wider area (Struebig et al., 2013), with higher than typical timber extraction rates. This is a diverse forest type from which we have recorded at least 175 species of bird and at least 50 species of amphibian from 26 sites (Sethi, Ewers, et al., 2020). Habitat ranges from areas of grass and low shrub, through logged forest to almost undisturbed primary forest.

### 2.2 Soundscape recording

**FIGURE 1:**
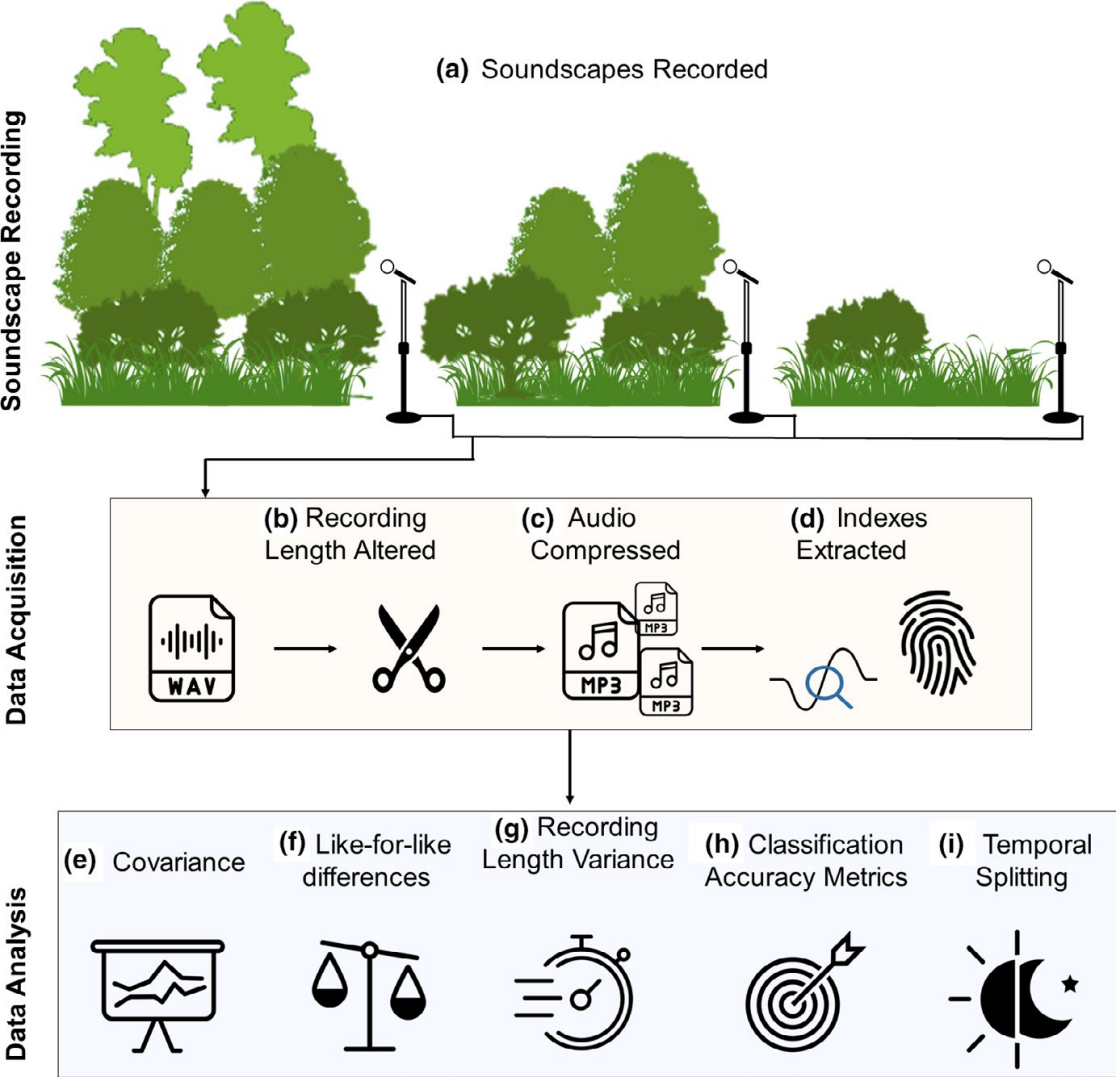
Experimental structure. Soundscape Recording: (a) Soundscapes from different forest structures in Malaysian Borneo are recorded. Data Acquisition: (b) Recording length is altered to 20‐, 10‐, 5‐, and 2.5‐min chunks; (c) all audio is compressed using nine lossy nine MP3 encoding techniques; (d) Analytical Indices and CNN Derived AudioSet Fingerprint are calculated from audio of all lengths and compressions. Data Analysis: (e) Index covariance is found per index type and correlation with maximum frequency is found; (f) like‐for‐like differences of indices calculated from compressed versus uncompressed counterparts are found; (g) intragroup variance compared for the recording lengths; (h) the indices of both types, lengths, and compressions are tested with a supervised random forest classification task; (i) the dataset is split into temporal sections and classification accuracy is found

Data were collected from three KFR sites representing a gradient in aboveground biomass (Figure 1a; AGB: Pfeifer et al., 2016): primary forest (AGB = 66.16 t.ha^−1^), logged forest (AGB = 30.74 t.ha^−1^), and cleared forest (AGB = 17.37t.ha^−1^) (Appendix S1: Supplementary 1). We recorded continuously from a single recorder for a mean of 72 hr at each site (range: 70 to 75) during February and March 2019 (Appendix S1: Supplementary 2a). No rain fell during the recording period, so no recordings were excluded due to confounding geophony (Zhang et al., 2016). In all three sites, we placed individual omnidirectional (Hill et al., 2018) recorders, which were attached to trees (~50 cm diameter and 1–2 m above the ground) and recorded 20‐min samples with no break period and stored them as uncompressed files (“raw,”.wav format) at 44.1kHz and 16 bits.

### 2.3 Compressing and resizing the raw audio

Continuous 20‐min recordings were first split into recordings with a length of 2.5, 5.0, and 10.0 min, using the python package *pydub* (Robert & Webbie, 2018; Figure 1b) resulting in 8, 4, and 2 times as many recordings, respectively. The audio was then converted to lossy MP3 format using the fre:ac LAME encoder (Kausch, 2019) under two standard LAME MP3 encoding techniques: constant bit rate (CBR) and variable bit rate (VBR) compressions (Figure 1c). CBR reduces the file size to a specified number of kilobits per second; VBR varies bitrate per second depending on the analysis of the acoustic content and a quality setting (0, highest quality, larger bitrate; 9 lowest quality, smaller bitrate). Since bitrates are not directly comparable between VBR and CBR—and because storage savings are often the principal driver of compression choices—we used compressed file size as our measure of compression level. We used VBR0 and CBR320, CBR256, CBR128, CBR64, CBR32, CBR16, and CBR8, which resulted in file sizes ranging between 41.6% (CBR320) and 1.04% (CBR8) of the original raw file size and some reductions in Nyquist frequency (Table 1). We do not consider lossless compression, as the storage capacity is much higher and the files are obligatorily the same postdecompression. Previous studies have also found that the lossless compressed audio is largely identical to raw audio (Linke & Deretic, 2020).

**TABLE 1:** Bitrate, percentage file size reduction, and maximum encodable frequency for the experimental compression levels

Compression level	Bit storage/s	% File size	Nyquist frequency (kHz)
RAW	Constant: 768 kb	100	22.05
VBR0	Variable: ~ 127–250 kb	Mean = 20.82 Range = 32.64–16.63	22.05
CBR320	Constant: 320 kb	41.6	22.05
CBR256	Constant: 256 kb	33.35	22.05
CBR128	Constant: 128 kb	16.67	22.05
CBR64	Constant: 64 kb	8.33	22.05
CBR32	Constant: 32 kb	4.16	11.025
CBR16	Constant: 16 kb	2.08	8
CBR8	Constant: 8 kb	1.04	4

### 2.4 Quantification of soundscapes using indices

#### 2.4.1 Analytical indices

We used the *seewave (ver 2.1.6)* (Sueur, Aubin, et al., 2008) and *soundecology (ver 1.3.3)* (Villanueva‐Rivera & Pijanowski, 2016) packages in R (ver 3.6.1; R Core Team, 2020) to extract 7 Analytical Indices (Figure 4d): Acoustic Complexity Index (ACI, calculated per minute and averaged), Acoustic Diversity Index (ADI), Acoustic Evenness (AEve), Bioacoustic Index (Bio), Acoustic Entropy (H), Median of the Amplitude Envelope (M), and Normalised Difference Soundscape Index (NDSI; Appendix S1: Supplementary 3). These have been shown to capture diel phases, seasonality, and habitat type (Bradfer‐Lawrence et al., 2019). These indices could not be calculated for all recordings due to file reading errors; however, this fault occurred in 0.3% of all recordings (Appendix S1: Supplementary 2b).

#### 2.4.2 AudioSet fingerprint

The audio was converted to a log‐scaled Mel‐frequency spectrogram after 16 kHz downsampling and then passed through the “VGG‐ish” Convolutional Neural Network (CNN) trained on the AudioSet database (Gemmeke et al., 2017; Hershey et al., 2017; Figure 1d). This generated a 128‐dimensional embedding and the 128 values in that embedding described the soundscape of given recording in an abstracted form or fingerprint. Similarly, as in the Analytical Indices, some recordings could not be analyzed by the AudioSet CNN; however, this was only in 0.2% of recordings (Appendix S1: Supplementary 2b).

### 2.5 Data analysis

#### 2.5.1 Impact of index selection: auto‐correlation

Analytical Indices often summarize similar features of a soundscape (e.g., dominant frequency and frequency bin occupancy): This overlap may reduce the descriptive scope of the ensemble. We compared the degree of pairwise correlation between the individual Analytical Indices and between the individual values of the AudioSet Fingerprint. We also compared how well each index/feature correlated with the Nyquist frequency (Figure 1e).

#### 2.5.2 Impact of compression: like‐for‐like differences

We used an adaption of Bland–Altman plots (Araya‐Salas et al., 2019; Vesna, 2009) to visualize the scaled difference (*D*) between raw (*I*_raw_) and compressed (*I*_com_) index values, as a percentage of the range of raw values *R*_raw_ (Figure 1f):

$$D=\frac{I_{\text{com}}‐I_{\text{raw}}}{R_{\text{raw}}}\times 100$$

*D* was not normally distributed (Appendix S1: Supplementary 5a), so median and interquartile ranges were reported. We determined that an index has been altered as a result of compression to be when: (a) the interquartile range of *D* did not include zero difference or (b) median *D* was more than ±5% of the *R*_raw_. We used Spearman rank correlation to test for a consistent trend in *D* with increasing compression. To reflect their common use cases, *D* for Analytical Indices was calculated from the univariate values, while for AudioSet Fingerprints—which is intended as a multidimensional metric—*D* was calculated separately for each dimension and then given as a mean of all 128 values.

#### 2.5.3 Impact of recording schedule: recording length

Recordings of longer length may have a reduced variance due to the smoothing of potentially important transient audio anomalies (such as nearby bird or cicada calls). We tested this by comparing the variance of the recording groups at different commonly used recording lengths. The index values are non‐normally distributed so we used Levene's test for homogeneity of variance (Figure 1g).

#### 2.5.4 Impact of parameter alteration on classification task

We used random forest classification models to assess how well the soundscapes were represented by each index type under each different experimental parameter, using the *RandomForest (ver 4.6‐14)* (Liaw & Wiener, 2002) package in R (Figure 1h). Models were trained on a 24‐hr period of data from each site and tested on the remaining 46 + h of audio. We used 2,000 decision trees to ensure accuracy had stabilized. The model was trained and tested separately for every combination of index type (Analytical Indices vs. AudioSet Fingerprint), compression level, and recording length. We determined the accuracy, precision, and recall of each combination.

#### 2.5.5 Impact of temporal subsetting

Soundscapes typically show considerable diel variation in both abiotic and biotic components. To assess the impact of this variance on model performance, we split our recordings into four 6‐hr sections centered on the key periods of Dawn (06:00), Noon (12:00), Dusk (18:00), and Midnight (00:00) and then further subdivided these into 3‐hr (8 sections) and 2‐hr (12 sections) blocks to test how further reductions affected the model (Figure 1i). We trained and tested the random forest model again on each of the temporally subset recordings, with each section used to build models individually, and determined accuracy, precision, and recall as before.

#### 2.5.6 Modeling the impact of index selection, compression, and recording length on the accuracy metrics

As the accuracy metrics are bound between 0% and 100%, we used a beta regression to model the relationship between each of the experimental parameters and performance metrics (Douma & Weedon, 2019). The model was built using the *betareg (ver 3.1‐3)* package in R (Cribari‐Neto & Zeileis, 2010). To avoid fitting issues when performance measures are exactly 1, we rescaled all performance measures using *m*′ = (*m* (*n*−1) + 0.5)/*n*, where *n* is the sample size (Smithson & Verkuilen, 2006). The model included pairwise interactions between file size, temporal subsetting, and recording length, and then all interactions of main effects and those pairwise terms with the index selection. We observed that variance in performance measures varied as an interaction of both index choice and a temporal subsetting (Appendix S1: Supplementary 8a), so tested the inclusion of these terms in the precision component of the model. We first treated recording length and temporal subsetting as factors, but also tested a model considering these as continuous variables. We found the Akaike information criterion (AIC) was markedly lower in a beta regression model using factors and including the precision component (Appendix S1: Supplementary 8b).

## 3 RESULTS

**FIGURE 2:**
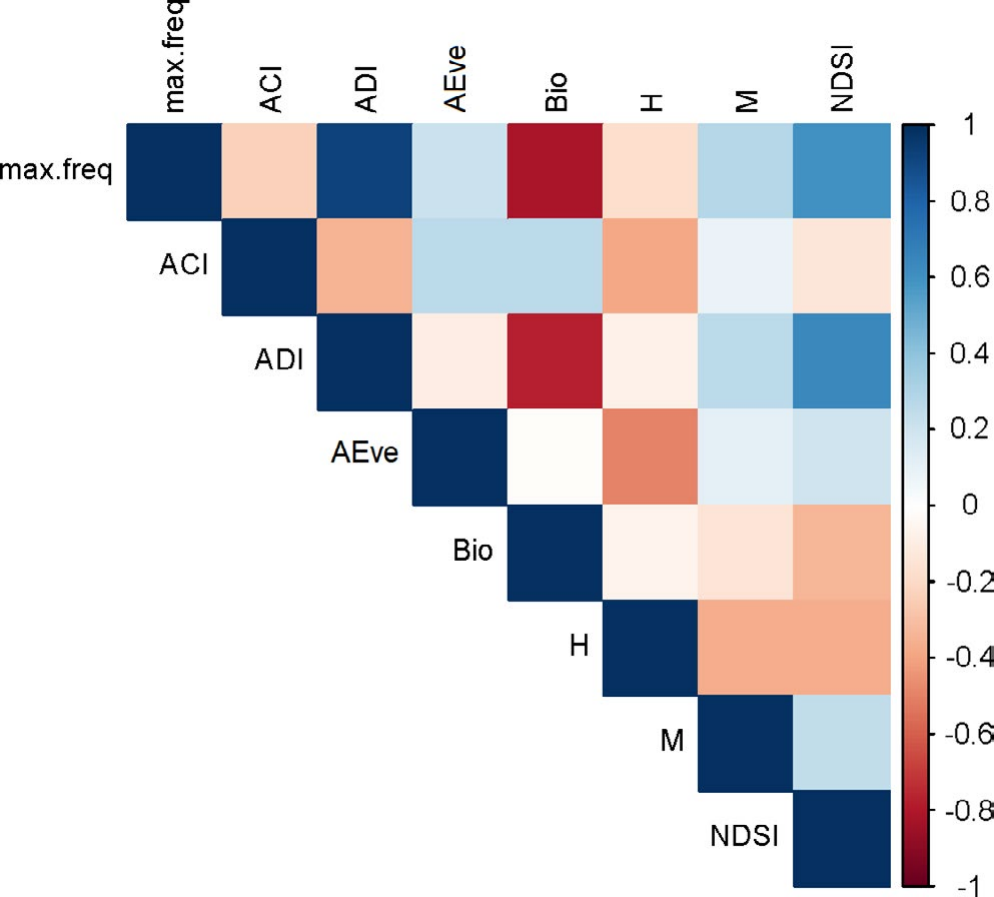
Pairwise Spearman correlation matrix for Analytical Indices (all recording lengths and all compressions) and maximum recordable frequency. The color scale shows rho values

Although Spearman pairwise correlations of Analytical Indices and Nyquist frequency were low on average (mean = 0.32, IQR = 0.22), we found some strongly correlated sets of indices (Figure 2). ADI, Bio and NDSI all show strong similarities and were closely correlated with maximum recordable frequency; AEve and H were also strongly correlated (Figure 2). Some features of the AudioSet Fingerprint correlated with each other and maximum frequency, but in general, these features were more weakly correlated (mean = 0.14, IQR = 0.18; figure in Appendix S1: Supplementary 4b).

### 3.1 Impact of compression

#### 3.1.1 Impact of compression: like‐for‐like differences

**FIGURE 3:**
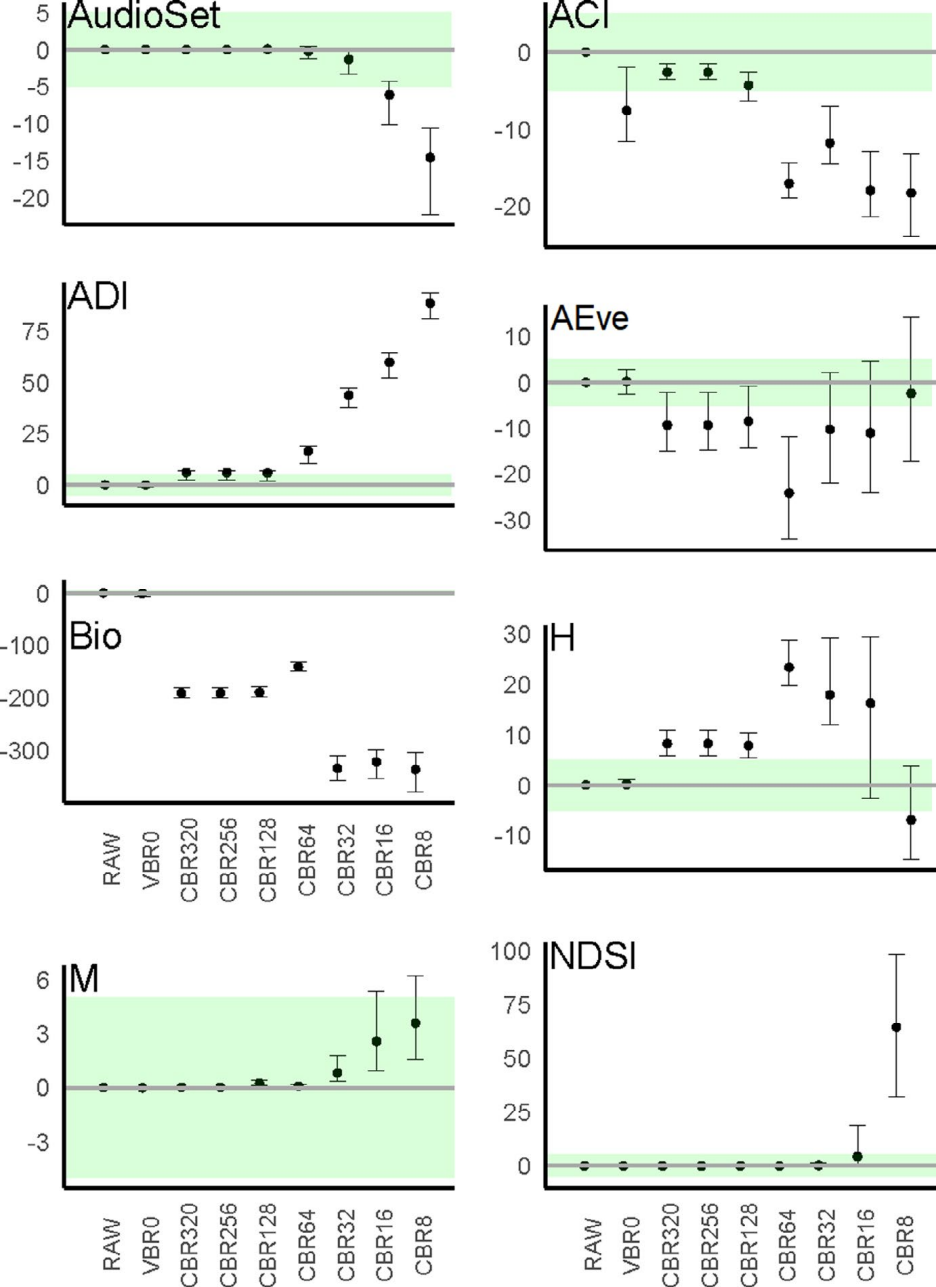
Scaled difference in acoustic indices from raw audio with increasing compression in 5‐min audio samples (see Appendix S1: Supplementary 4 for 2.5‐ and 10‐ and 20‐min examples). The horizontal green region shows the ±5% *D*. Dots and whiskers show the median and interquartile range of *D* from different indices under increasing levels of compression

Both index types showed both differences under compression and clear trends with increasing compression (Figure 3; confirmed with Spearman's rank correlation, all *p* < .001; Appendix S1: Supplementary 5b). The mode of response showed three broad qualitative patterns, illustrated here using results from the 5‐min audio sample (other recording lengths in Appendix S1: Supplementary 5a). (a) Indices which were only affected above a threshold level of compression (AudioSet Fingerprint: CBR16; M: CBR32; and NDSI: CBR8). These indices typically showed low absolute *D* (median *D* typically <15%). (b) AEve and H showed the largest differences at an intermediate compression (CBR64) and relatively low absolute differences (median *D* typically <30%). (c) The remaining indices showed a variety of responses: ADI showed a monotonic response above a threshold, ACI showed changes up to CBR64 and then stabilizes, and Bio showed a stepped pattern of increase. However, all three showed increasing and large changes in absolute D (median *D* often >75%) with increasing compression.

#### 3.1.2 Impact of recording schedule: recording length

Three out of seven (43%) of the Analytical Indices (ADI, AEve, and H) and a smaller proportion of the AudioSet Fingerprint values (46 out of 128; 36%) were found to have nonhomogeneous variance in groups of different recording lengths (*p* < .05, Levene's test for homogeneity of variance; Appendix S1: Supplementary 6b).

### 3.2 Impact of index selection

Confirming prior findings (Sethi, Jones, et al., 2020), we showed that habitat classifiers derived from 5‐min recordings using raw audio showed higher accuracy for AudioSet Fingerprint (93.8%) than Analytical Indices (80.9%; Table 2). This advantage held across all recording lengths and performance metrics with performance gains of around 12%–13% in accuracy, precision, and recall (Appendix S1: Supplementary 7b).

**TABLE 2:** Confusion matrices from random forest classifiers trained on AudioSet Fingerprint (a, c) and Analytical Indices (b, d) using uncompressed raw audio (a, b) and highly compressed CBR8 audio (c, d)

Observed	AudioSet Fingerprint			Observed	Analytical Indices		
	Predicted				Predicted		
**(a) Raw**	Cleared	Logged	Primary	**(b) Raw**	Cleared	Logged	Primary
Cleared	585	9	11	Cleared	484	67	49
Logged	11	508	44	Logged	97	421	46
Primary	17	14	521	Primary	9	61	486
**(c) CBR8**	Cleared	Logged	Primary	**(d) CBR8**	Cleared	Logged	Primary
Cleared	585	3	17	Cleared	484	23	98
Logged	2	488	73	Logged	9	379	175
Primary	11	53	488	Primary	9	115	428

Compression decreased accuracy for both AudioSet Fingerprint (CBR8: 90.8%) and Analytical Indices (CBR8: 75.1%; Table 2). Classifiers trained on *compressed* AudioSet Fingerprint, however, still outperformed those trained on *uncompressed* Analytical Indices. For both index types, this reflected a decreased ability to differentiate logged and primary forest. Interestingly, classifiers from both index types showed better discrimination between cleared land and logged forest under strong compression. These patterns were repeated across recording lengths (Appendix S1: Supplementary 5a).

#### 3.2.1 Impact of temporal subsetting

**FIGURE 4:**
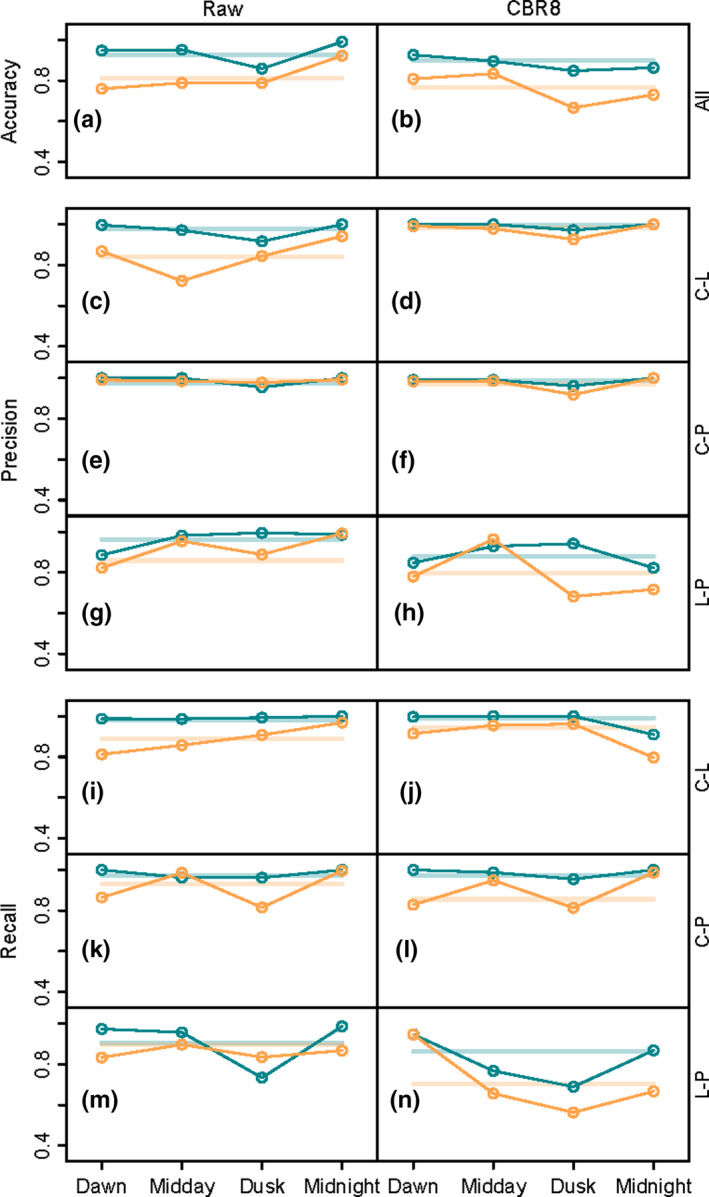
Classification model performance as a function of temporal sectioning (*x*‐axis), compression (raw audio, left column; CBR8 compression, right column) and index choice (AudioSet Fingerprint: blue; Analytical Indices: orange). Pale horizontal lines show performance without temporal sectioning. Precision and recall are partitioned into pairwise performance by site (C, cleared forest; L, logged forest; P, primary forest)

Temporally subsetting poses a trade‐off as when diel variation is reduced, so too are the recording hours available for analysis. Temporally subsetting the day into quarters (Figure 4) yielded a largely unpredictable effect on accuracy, precision, and recall. There were clear differences in discrimination between pairs of sites. Notably comparing cleared and primary forest had the highest precision across each temporal subset, index choice, and compression (Figure 4e,f), but the recall was not markedly different from other pairs (Figure 4 k,l). Temporal windows did not generally help discriminate between logged and primary forest (Table 2, Figure 4g,h,m,n), and the performance difference between AudioSet Fingerprints and Analytical Indices was largely maintained.

#### 3.2.2 Combined effects of parameter alterations on classification performance

**FIGURE 5:**
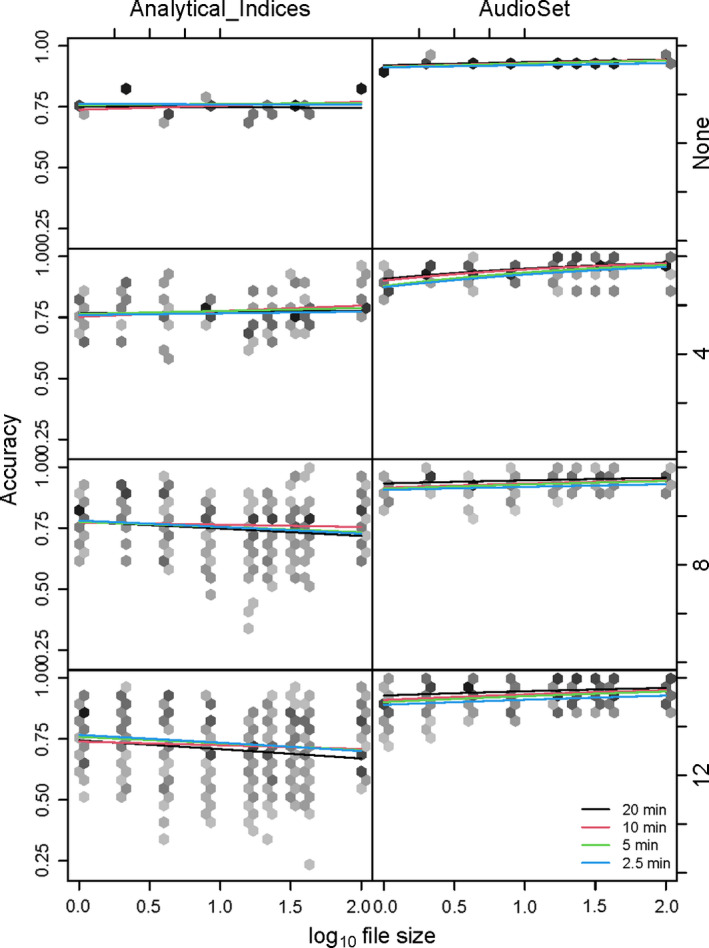
Classifier accuracy model predictions as a function of file size (*x*‐axis), index type (columns), temporal subsetting (rows), and frame size (colors, see legend). Hexagon binning is used to show the distribution and density of the underlying data

Confirming prior findings (Sethi, Jones, et al., 2020), our model has demonstrated that performance measures were consistently higher when classifiers are trained on the AudioSet Fingerprint, rather than Analytical Indices (accuracy: +16.9% (*z* = 10.38_1799_ *p* < .001), precision: +15.5% (*z* = 9.717_1799_ *p* < .001), recall: +16.9% (*z* = 10.22_1799_ *p* < .001), full model outputs Appendix S1: Supplementary 9C). Index type was by far the largest contributor to model accuracy (Table 3), although there was some effect of temporal subsetting, compression level, and frame size. Despite the considerable impact of compression level on index values, it appeared to have a minor effect on model accuracy (Figure 5, Table 3). The effect of frame size appeared to increase as the days were cut into smaller temporal subsections; however, this effect was small compared with the contribution of index type (Figure 5). Temporal subsetting appeared to have minimal effect on the accuracy of the AudioSet Fingerprint classifier, which kept consistently high (70%–100%; Figure 5). The classifier trained on Analytical Indices, however, became much more unpredictable when temporal subsetting is used (20%–100%; Figure 5).

**TABLE 3:** ANOVA table for the model terms in the beta regression model of the accuracy data (Significance: ***p < .001, **p < .01, *p < .05. Equivalent tables for precision and recall in Appendix S1: Supplementary 9C)

	*df*	*χ* ^2^
log10(File Size)	1	26.2128***
Temporal Subsetting	3	31.6818***
Frame Size	3	15.7820**
Index Type	1	2,985.9825***
log_10_(File Size): Temporal Subsetting	3	18.0278***
log_10_(File Size): Frame Size	3	2.9280
Temporal Subsetting: Frame Size	9	6.3156
log_10_(File Size): Index Type	1	59.0065***
Temporal Subsetting: Index Type	3	7.1061
File Size: Index Type	3	36.2699***
log_10_(File Size): Temporal Subsetting: Index Type	3	13.0715**
log_10_(File Size): Frame Size: Index Type	3	0.8071
Temporal Subsetting: Frame Size: Index Type	9	7.1524

## 4 DISCUSSION

Ecoacoustics is a new and rapidly expanding field of ecology, with great power to describe ecological systems (e.g., Sethi, Jones, et al., 2020), but methodological choices have proliferated that have poorly known impacts on ecoacoustic analysis. We have shown that the choice of acoustic index is key and confirm (Sethi, Jones, et al., 2020) that a multidimensional generalist classifier (AudioSet Fingerprint) outperforms more traditional Analytical Indices regardless of the levels of audio compression or recording schedule.

Analytical Indices have been constrained to a limited set of features within soundscapes, leading to high degrees of correlation. For example, ADI, AEve, and H indices are all summaries of the evenness of frequency band occupancy (Sueur, Aubin, et al., 2008; Villanueva‐Rivera et al., 2011). This nonindependence can further decrease the dimensionality of suites of Analytical Indices, which are already typically small. Here, we use just the mean values of Analytical Indices, but other studies have incorporated both the mean and standard deviation (Bradfer‐Lawrence et al., 2019), which provides further dimensionality. Although the AudioSet Fingerprint clearly benefits from a large number of relatively uncorrelated acoustic features, most Analytical Indices have the advantage of being designed to capture ecologically relevant aspects of the soundscape.

Compression affected the quantification of all indices in both index types (Figure 3) and—although the qualitative patterns were noisy—the groupings seen may reflect the underlying algorithms. The apparent threshold for AudioSet Fingerprint at CBR16 may be due to the obligatory loss in audio quality before samples pass to the CNN used to generate the AudioSet Fingerprint. The audio was downsampled to 16 kHz and then presented as a mel‐shifted spectrogram, which increases sensitivity in frequency ranges relevant to human hearing, akin to those frequencies favored in commercial compression. Coupled with its variable quality training set (YouTube Videos), these factors may predispose AudioSet Fingerprint to perform as well with high‐quality audio as with intermediate and low‐quality MP3s.

The M and NDSI were also largely unaffected by compression until the frequency range is reduced. When mp3 audio is compressed below 32 kb/s the audio swaps from being encoded as MPEG‐1 Audio Layer III (which supports max frequency of 16–24 kHz) to MPEG‐2 Audio Layer III (max: 8–12 kHz), this change in format results in the removal of signals beyond the cutoff frequency threshold. Further reduction is seen where at CBR8 when encoding changes again to MPEG‐2.5 Audio Layer III (max: 4–6 kHz). The M index is explicitly a measure of amplitude (Sueur et al., 2014) and is largely unaffected until downsampling reduces amplitude. Similarly, NDSI measures the proportion of sound in biophonic versus anthropophonic frequency bands: As downsampling progressively eliminates sounds within the frequency range (2–11 kHz) containing most biophony, NDSI is known to increase (Kasten et al., 2012). The ADI index also shows a marked increase in the magnitude of the difference at higher rates of compression (CBR64); however, a small but significant difference can be observed from CBR256. The ADI index measures the spread of frequencies above a certain loudness threshold, the effect of compression on ADI, may therefore suggest that certain high‐frequency bands are dominant in this soundscape.

AEve and H, both of which describe the spread and evenness of amplitude over the full range of frequencies, showed a gradual increase in *D* that reversed when the Nyquist frequency reduced. The two measures differ in measuring dominance (AEve: Villanueva‐Rivera et al., 2011) and evenness (H: Sueur et al., 2014) across bands but may share a common explanation. In both cases, compression preferentially removed amplitude from some bands, initially decreasing evenness but downsampling removes bands entirely, possibly restoring a more even distribution.

ACI and Bio both shared a dependence on high frequency or quieter sounds and were generally most severely affected by compression. ACI measures frequency band‐dependent changes in amplitude over time (Pieretti et al., 2011) and is reduced when there is minimal variation between time steps. Loss of “masked” sounds under low compression and then 16–24 kHz sound under CBR16 may reflect the loss of ecoacoustic temporal variation: This band includes the calling range of many invertebrates, birds, mammals, and amphibians (Browning et al., 2017). The Bio index similarly quantifies the spread of frequencies in the range 2 kHz–11 kHz, all relative to the quietest 1 kHz band (Boelman et al., 2007): Loss of quiet frequency bands, therefore, make it uniquely sensitive to compression. Despite both of these indices incurring alterations 200% larger than the uncompressed range, the Analytical Indices classifier accuracy still showed robustness to compression, perhaps suggesting these indices are less important for classification than the others. Bradfer‐Lawrence et al. (2019) have already shown that the Bio index contributes little additional power when classifying soundscapes, but found that ACI was the strongest individual contributor in this suite of indices (Bradfer‐Lawrence et al., 2019). Our findings suggested this ranking may not be consistent across different levels of compression.

Our findings reflect those of an earlier study that explored the effect of mp3 compression (VBR0 and CBR128) on indices describing specific bird calls (Araya‐Salas et al., 2019). They found that compression did not cause a systemic deviation in all indices, but rather indices designed to capture extreme frequencies were less precise after compression, particularly with VBR encoded files (Araya‐Salas et al., 2019). While some of these principles are present in our findings, the use of a wider range of compressions has allowed us to develop a more complete description of the action of compression on soundscape indices.

We found that even the highest rate of compression caused a comparatively small reduction in the overall accuracy of the classification task (5.8% and 3% for Analytical Indices and the AudioSet Fingerprint, respectively, for the 5‐min recordings without temporal subsetting). In both cases, the reduction in accuracy was explained by a higher degree of overlap between primary and logged forests. When audio is compressed, the whole signal is altered but higher frequencies and quieter sounds are more severely altered and reduced than others (Sterne, 2012). Higher and quieter frequencies (akin to specific animal vocalizations) may therefore be more important for separating logged and primary—but less so for discerning cleared from other forest types (which may be more dependent on overall amplitude). These proportionally small differences, while somewhat reassuring, should be considered with caution they may be due to the large differences in habitat structure among our three habitat classes. Combining this with our relatively small sample size, we would like to emphasize that these findings may therefore not be generalizable to areas of more closely related forest.

Both Analytical Indices and AudioSet Fingerprint had similar changes in variance as a result of recording length. Transient vocalizers are therefore likely somewhat important in the determination of the AudioSet Fingerprint and variable importance in some Analytical Indices. The ACI index was not impacted by recording length despite specifically quantifying how the soundscape changes over time (Pieretti et al., 2011). The ADI, AEve, and H all did incur an alteration in variance as recording length changed; interestingly, these indices do not consider any temporal value but rather just the spread of frequency (Sueur, Pavoine, et al., 2008; Villanueva‐Rivera et al., 2011), indicating that transient calls akin to short‐term anomalies in frequency are perhaps lost when recording windows are altered.

Finally, we found that subsetting audio data temporally and analyzing them separately had an unpredictable impact on classification accuracy, with the AudioSet Fingerprint classifier staying consistently high while the Analytical Indices classifier was returning accuracies anywhere between 20% and 100%. Temporal subsetting can reduce the impact of diel variation on analyses but poses a trade‐off as it reduces the amount of data used to train the classifier. Analytical Indices may perform better over longer recording periods as >120 hr of recordings are required for Analytical Indices to stabilize (Bradfer‐Lawrence et al., 2019), yet in our study, we had just 70–75 hr of recordings per site. Overall we found that compression, frame size, and temporal subsetting caused a small decrease in classifier accuracy, with the largest overall contributor being the choice of AudioSet Fingerprinting over Analytical Indices. The AudioSet Fingerprint classifier, temporally sectioned, and trained on just 2 hr of data was able to, on average, outperform the Analytical Indices classifier trained on the full 24 hr.

## 5 RECOMMENDATIONS AND CONCLUSION

This study was designed to compare distinct forest types in Malaysian Borneo, and the recording periods used are relatively small. Based on the results of this study, we provide the following four recommendations; however, effort should be made to ensure they are generalizable to the desired area of deployment:

1. We provide additional evidence for the viability and stability of AudioSet Fingerprinting rather than Analytical Indices when classifying soundscapes.
2. Lossless compression is always desirable but if data storage/transmission become a bottleneck to a study, we advise using the VBR (quality = 0) MP3 encoder if using Analytical Indices, which will reduce the file size to roughly 23% of the original while having minimal impact on indices (other than ACI). The AudioSet Fingerprint, however, is more robust to compression and so can tolerate a minimum compression encoding of CBR64 (8% of the original file size) without significant effect.
3. If further compression is a necessity, use indices which describe the general energy of the system rather than those which are dependent on high frequency or quieter sounds, such as ACI.
4. Temporal subsetting may be a useful alternative for capturing soundscape descriptors with AudioSet Fingerprinting when data storage costs are a bottleneck. However, temporal subsetting should be used with caution when using Analytical Indices owing to the variation in classification accuracy, precision, and recall.

There exists a trade‐off between the quality and volume of data that can be stored in ecoacoustics. We have investigated the impact of compression along a gradient of habitat disturbance, providing evidence that compressed audio can be used without severely affecting either of the index type. The ability to use compression may reduce experimental costs, remove bottlenecks in study design, and help remote ecoacoustic recorders reach true autonomy. Moreover, by providing a quantified description of how individual indices, and more broadly grouped index categories, respond to compression, we have enabled comparisons to be drawn between studies of compressed and noncompressed audio. Increasing comparability of studies will become progressively important as global ecoacoustic databases, and recording sites grow and open up novel opportunities to explore datasets across huge temporal and geographic scales.

## CONFLICT OF INTEREST

No conflict of interest to declare.

## AUTHOR CONTRIBUTIONS

**Becky E. Heath:** Conceptualization (lead); Data curation (lead); Formal analysis (equal); Funding acquisition (equal); Investigation (lead); Methodology (equal); Project administration (lead); Visualization (equal); Writing‐original draft (lead); Writing‐review & editing (equal). **C. David L. Orme:** Data curation (equal); Formal analysis (equal); Supervision (equal); Visualization (equal); Writing‐review & editing (equal). **Sarab S. Sethi:** Conceptualization (supporting); Data curation (equal); Formal analysis (supporting); Methodology (equal); Writing‐review & editing (equal). **Robert M. Ewers:** Conceptualization (equal); Methodology (equal); Project administration (equal); Writing‐review & editing (equal). **Lorenzo Picinali:** Conceptualization (equal); Formal analysis (equal); Methodology (equal); Project administration (equal); Supervision (equal); Writing‐review & editing (equal).

### OPEN RESEARCH BADGES

This article has earned an Open Data Badge for making publicly available the digitally‐shareable data necessary to reproduce the reported results. The data is available at AudioSet/ Analytical Index Data: https://doi.org/10.5281/zenodo.5153193. Raw Audio Files: https://doi.org/10.5281/zenodo.5159914. Data Analysis Repo: https://github.com/BeckyHeath/Experimental‐Variation‐Ecoacoustics‐Analysis‐Scripts.

## Supporting information

Appendix S1

## Data Availability

Acoustic Data: Available at 10.5281/zenodo.5159914. Analytical Indices/AudioSet Fingerprint Data: Available at 10.5281/zenodo.5153193. Analysis Scripts: Available on Github at https://github.com/BeckyHeath/Experimental‐Variation‐Ecoacoustics‐Analysis‐Scripts (made public after publication).
