# Supplementary material for: How index selection, compression, and recording schedule impact the description of ecological soundscapes
Source: Ecol Evol. 2021 Aug 26;11(19):13206–17. doi: 10.1002/ece3.8042 (PMC8495811; doi:10.1002/ece3.8042)
Supplement: Supplementary file 1 — Appendix S1 [file ECE3-11-13206-s001.docx]

**SUPPLEMENTARY V4**

**The Impact of Index Selection, Compression Level, and Recording Schedule on the Description of Ecological Soundscapes**

Contents:

1 – AGB Information

2 - Recording Period Information

2a – Recording Time

2b – Number of Recordings per condition

3 – Analytical Index Further Descriptions

4 – Impact of Index Selection: Index Autocorrelation (Figure 2)

4a – Analytical Indices

4b – AudioSet Fingerprint

5 – Impact of Compression: Like-for-Like Differences (Figure 3)

5a – *D* Normality Testing, Make sure the figures are right

5b – Figure Alternatives

5c – Model Outputs

6 – Impact of Recording Schedule: Recording Length

6a – Levene’s test for Homogeneity of Variance

7 – Impact of Index Type: Confusion Matrices Raw vs. CBR8 (Table 2)

7a – Alternate Matrices

7b – Accuracy/Precision/Recall for

8 – Impact of Temporal Splitting (Figure 4)

8a – Alternate Figures

9 – Synthesis: Beta Regression Modelling the Contribution of all Parameters (Figure 5)

9a – Data Variance (*ϕ* component)

9b – AIC values for models

9c – Alternate Figures (Precision and Recall)

9d – Beta Regression and Linear Model Output Statistics

10 – References

**1 - Above Ground Biomass**

GPS Location +/- 1.11km (0.01°) to longitude and latitude

| **Location** | **Co-Ordinates** | **AGB (t.ha^-1^)** |
| --- | --- | --- |
| **Primary** | **117.53513, 4.66443** | **77.8151982458024** |
| Primary | 117.53513, 4.65443 | 54.9446402261185 |
| Primary | 117.52513, 4.66443 | 87.2290056404617 |
| Primary | 117.52513, 4.65443 | 3.18829485141835 |
| Primary | 117.53513, 4.67443 | 49.5375612006372 |
| Primary | 117.54513, 4.66443 | 116.326089963978 |
| Primary | 117.54513, 4.67443 | 74.0737999980781 |
| **Logged** | **117.58118, 4.69372** | **13.3752792205534** |
| Logged | 117.58118, 4.68372 | 4.77470660981513 |
| Logged | 117.57118, 4.69372 | 61.6814645965346 |
| Logged | 117.57118, 4.68372 | 67.7614569739226 |
| Logged | 117.58118, 4.70372 | 42.4952102365264 |
| Logged | 117.59118, 4.69372 | 18.5855492747789 |
| Logged | 117.59118, 4.70372 | 6.5248825548366 |
| **Cleared** | **117.59141, 4.70272** | **9.85390659254801** |
| Cleared | 117.59141, 4.69272 | 7.9067100668437 |
| Cleared | 117.58141, 4.70272 | 48.796127019185 |
| Cleared | 117.58141, 4.69272 | 3.96428134965722 |
| Cleared | 117.59141, 4.71272 | 5.65620070374987 |
| Cleared | 117.60141, 4.70272 | 38.4582510289304 |
| Cleared | 117.60141, 4.71272 | 6.92104474036953 |

Averages

**Primary:** 66.16

**Secondary:** 30.74

**Logged:** 17.37

**2 – Recording Period Information**

2a – Total Recording Time

| Site | Recording Period  (Feb-March 2019) | Hours and Minutes in rec period | Recording number (20min) |
| --- | --- | --- | --- |
| Cleared | 26^th^ 8:40- 1^st^ 11:07 | 74h 40min | 224 |
| Logged | 27^th^ 10 – 2^nd^ 9:00 | 71h 00min | 213 |
| Primary | 26^th^ 11 – 1^st^ 9:07 | 70h 20min | 211 |

Total Recoding Hours: 216h

Total Raw 20min Recordings: 648

2b – Number of Recordings Used per Condition

| Compression | Frame Size | AudioSet Readings | Analytical Index Readings |
| --- | --- | --- | --- |
| Raw | 20min | 647 | 645 |
| CBR320 | 20min | 648 | 646 |
| CBR256 | 20min | 648 | 647 |
| CBR128 | 20min | 648 | 648 |
| CBR64 | 20min | 647 | 648 |
| CBR32 | 20min | 648 | 648 |
| CBR16 | 20min | 648 | 646 |
| CBR8 | 20min | 648 | 648 |
| VBR0 | 20min | 648 | 645 |
| Raw | 10min | 1292 | 1292 |
| CBR320 | 10min | 1292 | 1292 |
| CBR256 | 10min | 1292 | 1291 |
| CBR128 | 10min | 1292 | 1292 |
| CBR64 | 10min | 1292 | 1291 |
| CBR32 | 10min | 1278 | 1292 |
| CBR16 | 10min | 1275 | 1291 |
| CBR8 | 10min | 1292 | 1292 |
| VBR0 | 10min | 1283 | 1291 |
| Raw | 5min | 2584 | 2584 |
| CBR320 | 5min | 2584 | 2583 |
| CBR256 | 5min | 2584 | 2578 |
| CBR128 | 5min | 2584 | 2579 |
| CBR64 | 5min | 2584 | 2584 |
| CBR32 | 5min | 2584 | 2584 |
| CBR16 | 5min | 2584 | 2584 |
| CBR8 | 5min | 2584 | 2584 |
| VBR0 | 5min | 2584 | 2583 |
| Raw | 2.5min | 5184 | 5176 |
| CBR320 | 2.5min | 5179 | 5160 |
| CBR256 | 2.5min | 5184 | 5180 |
| CBR128 | 2.5min | 5183 | 5168 |
| CBR64 | 2.5min | 5184 | 5164 |
| CBR32 | 2.5min | 5189 | 5168 |
| CBR16 | 2.5min | 5184 | 5183 |
| CBR8 | 2.5min | 5184 | 5168 |
| VBR0 | 2.5min | 5184 | 5156 |
| TOTAL | | 87,329 | 87,211 |
| Files Lost: | | 151 | 269 |

Theoretical Total: 87,480

Lost Files: 0.17% of files were lost in AudioSet Calculation, 0.31% of files were lost in Analytical Index Calculation – Mostly in the 2.5-minute set

**3 – Analytical Index Meanings**

*The below table was adapted from Bradfer‐Lawrence et al., 2019, provided here with permission*

| Index and Acronym | Measures | High Value Indicates | Low Value Indicates | Reference |
| --- | --- | --- | --- | --- |
| Acoustic Complexity Index (ACI) | Frequency band dependant changes in amplitude over time. A measure to designed to quantify acoustic irregularity typical of birdsong. | Storms and intermittent raindrops.  High levels of avian vocalisations and insect stridulation | Constant noise filling the whole spectrogram e.g. cicadas or wind | (Pieretti et al., 2011) |
| Acoustic Diversity Index (ADI) | A frequency band dependant measure of the proportion of signals above -50dBFS, ADI is calculated from the Shannon index of each band | High levels of geophonic or anthrophonic noise  Very quiet recordings with a minimal signal in any frequency band | The dominance of a particular frequency band such as insect noise | (Villanueva-Rivera et al., 2011) |
| Acoustic Evenness  (AEve) | A frequency band dependant measure of the proportion of signals above -50dBFS, AEve calculated from the Gini index of each band | The reverse of ADI, the dominance of a narrow frequency band relating to insect noise | Large amounts of geophony or near silence  Sometimes acoustically saturated soundscapes | (Villanueva-Rivera et al., 2011) |
| Bioacoustic Index (Bio) | A measure of both amplitude and number of occupied frequency bands between 2 and 11kHz, relative to the quietest 1kHz frequency band | The greater dissimilarity between quiet and loud bands  Loud tonal insect noise such as cicadas | No sound in the given range, although some biophony does occur outside of this range | (Boelman et al., 2007) |
| Acoustic Entropy (H) | Measures spread of amplitude across frequency bands and/or time steps. Between 1 and 0, (diffuse and concentrated sound respectively) | Near silent recordings and faint bird calls  Rain and wind | When insect noise dominates a single frequency band | (Sueur et al., 2008) |
| Median of the Amplitude Envelope (M) | Measure of recording amplitude | High levels of geophony, particularly storms | Quiet recordings | (Sueur et al., 2014) |
| Normalised Difference Soundscape Index (NDSI) | Ratio of anthrophony (1-2kHz) and biophony(2-11kHz) | High levels of biophony, particularly insects | When insect biophony dominates 1-2kHz range instead | (Kasten et al., 2012) |
|  |  |  |  |  |

**4 – Index Autocorrelation (Figure 2)**

4a – Analytical Indices (same as in main text):


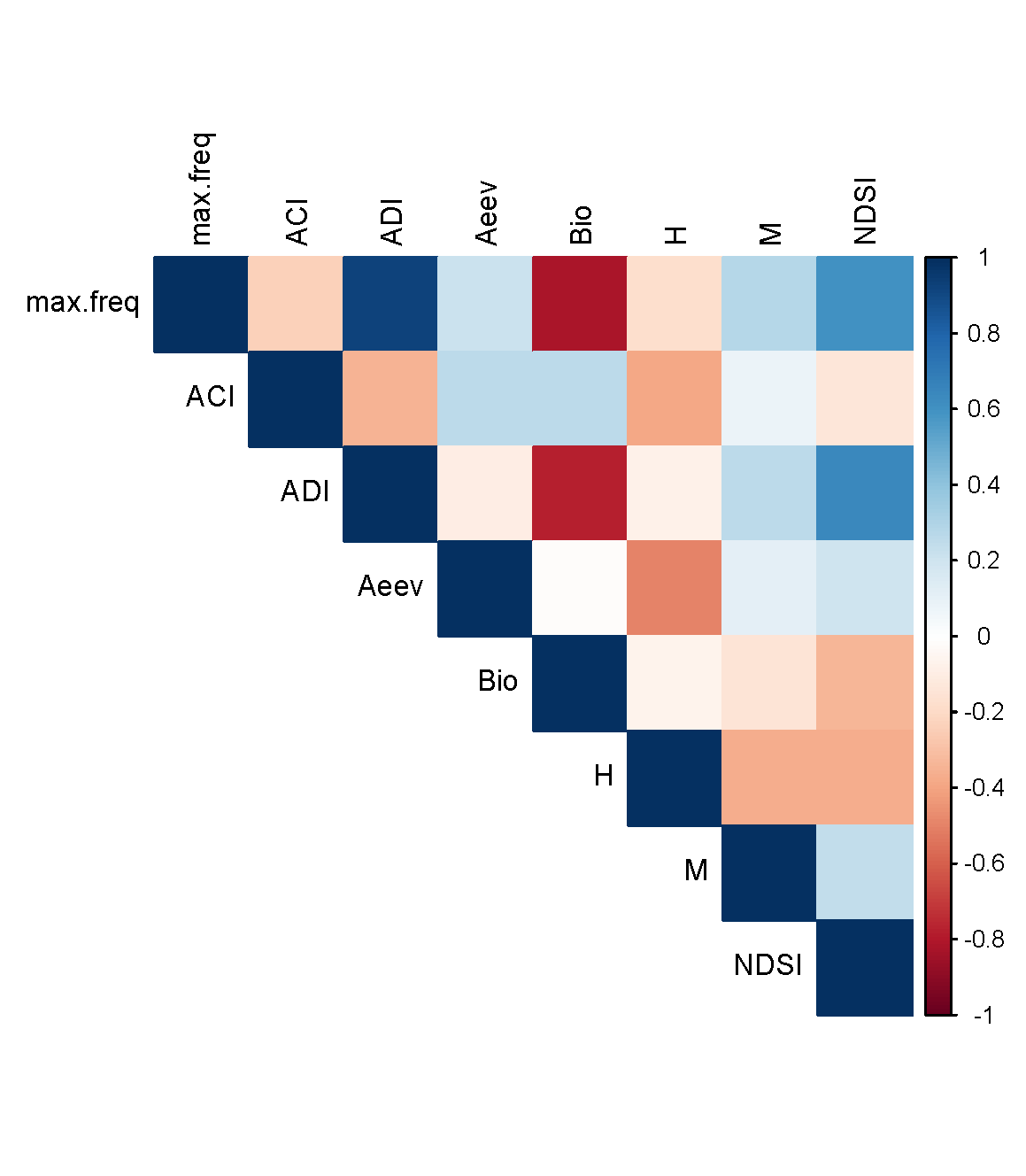


4b – AudioSet Indices (features in order: max.freq, feat1, feat2…feat128)


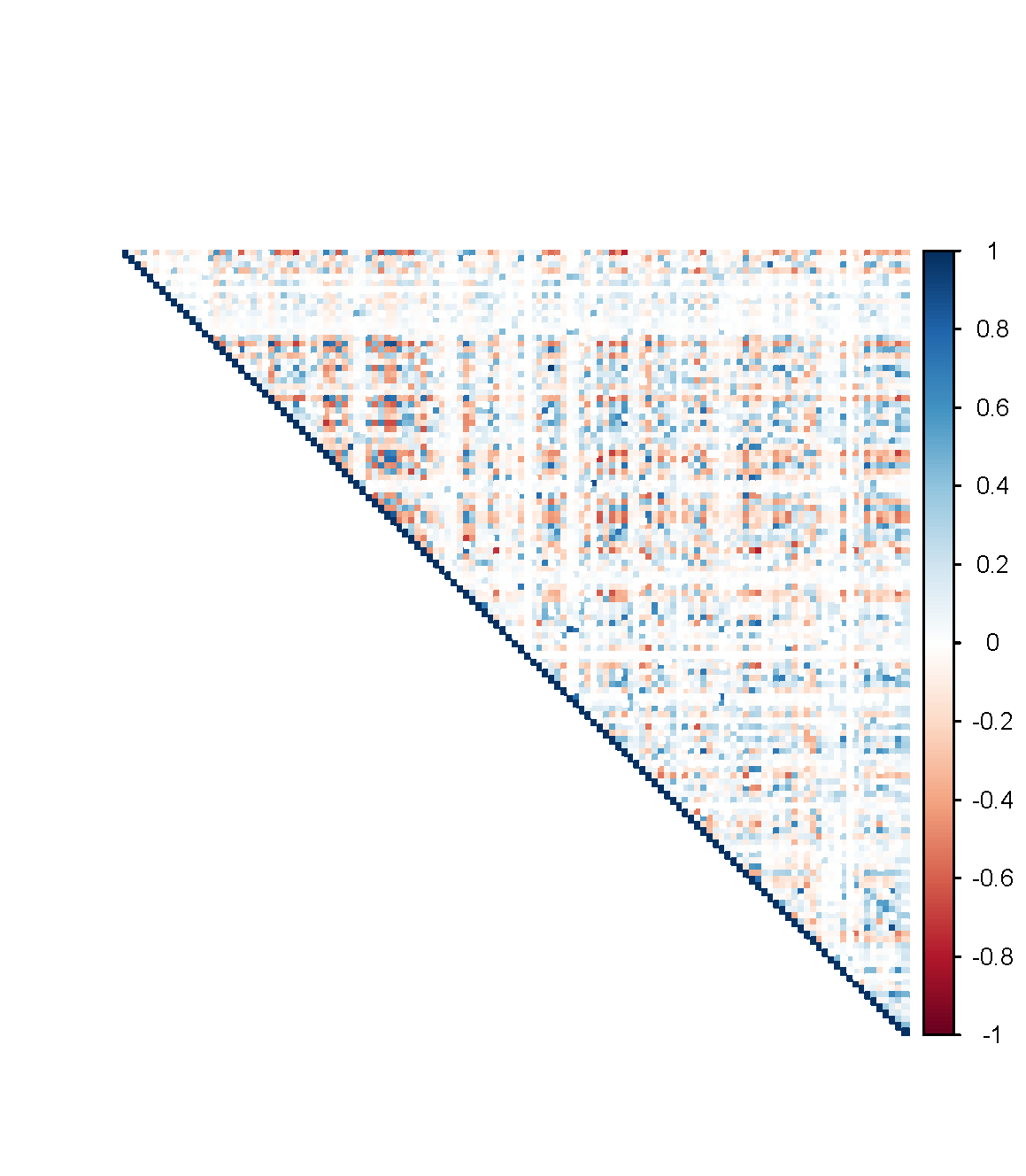


**5 – Impact of Compression: Like-for-Like Differences (Figure 3)**

5a – *D* Normality Testing, Make sure the figures are right

**AudioSet Fingerprint**


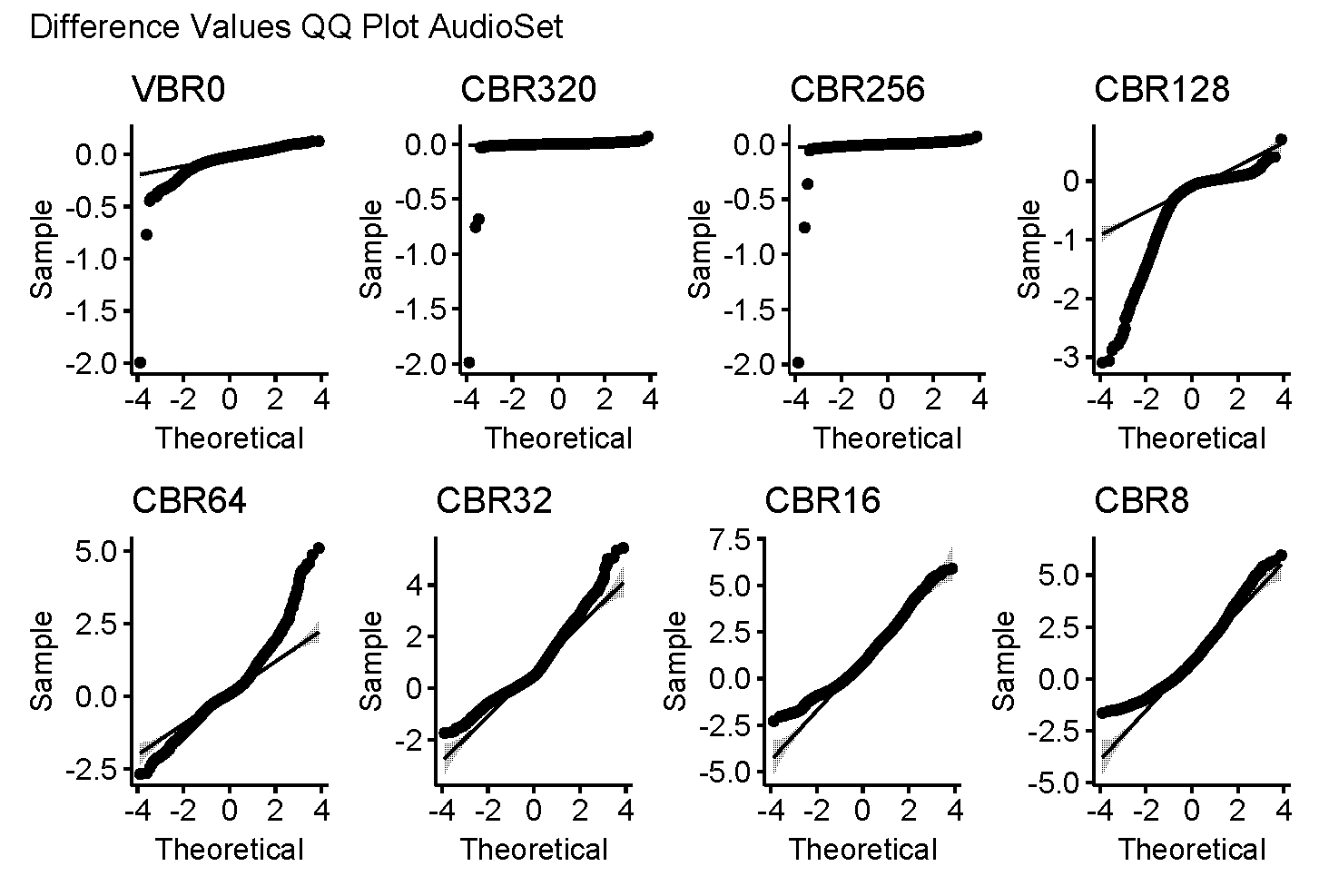


**Analytical Indices**


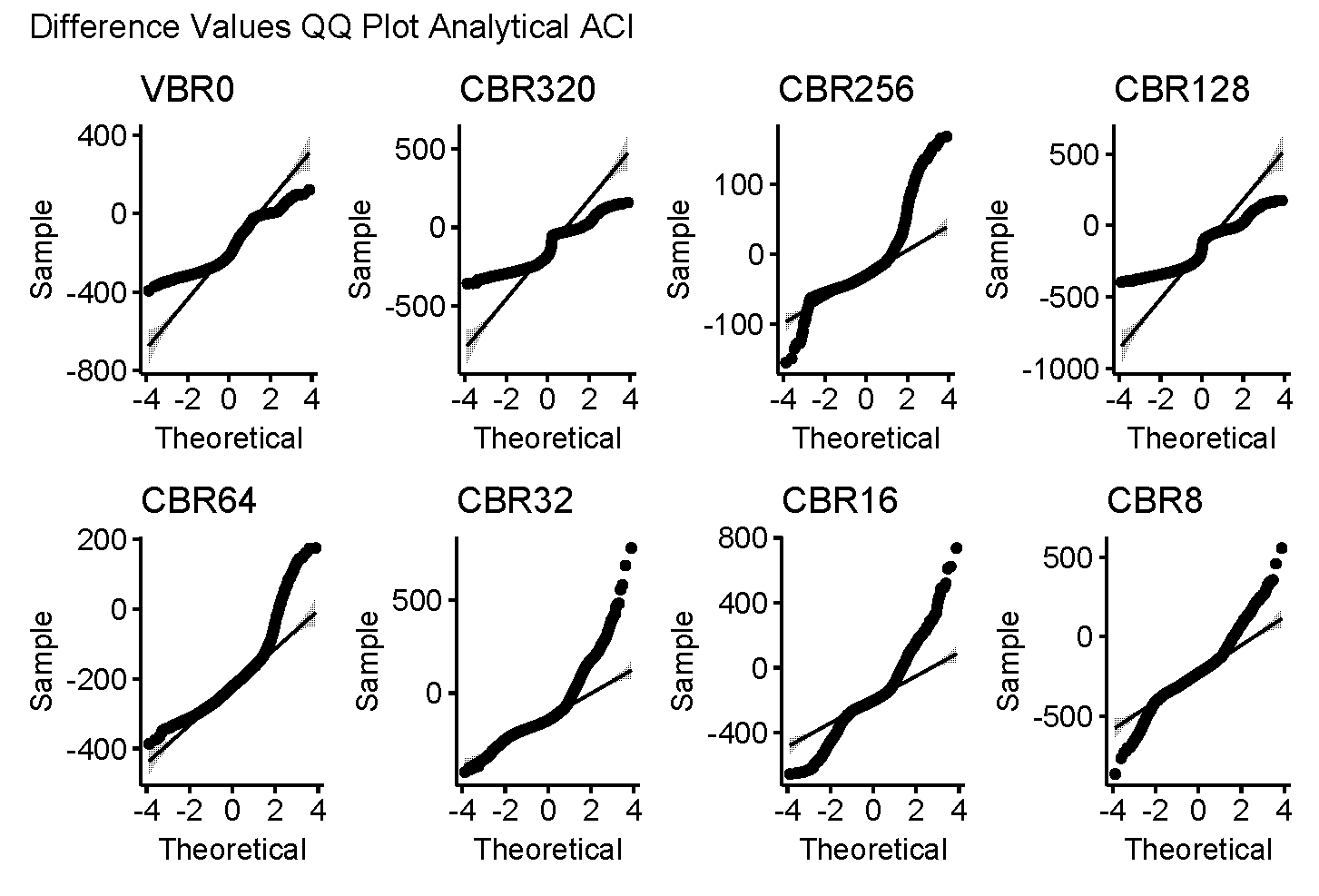


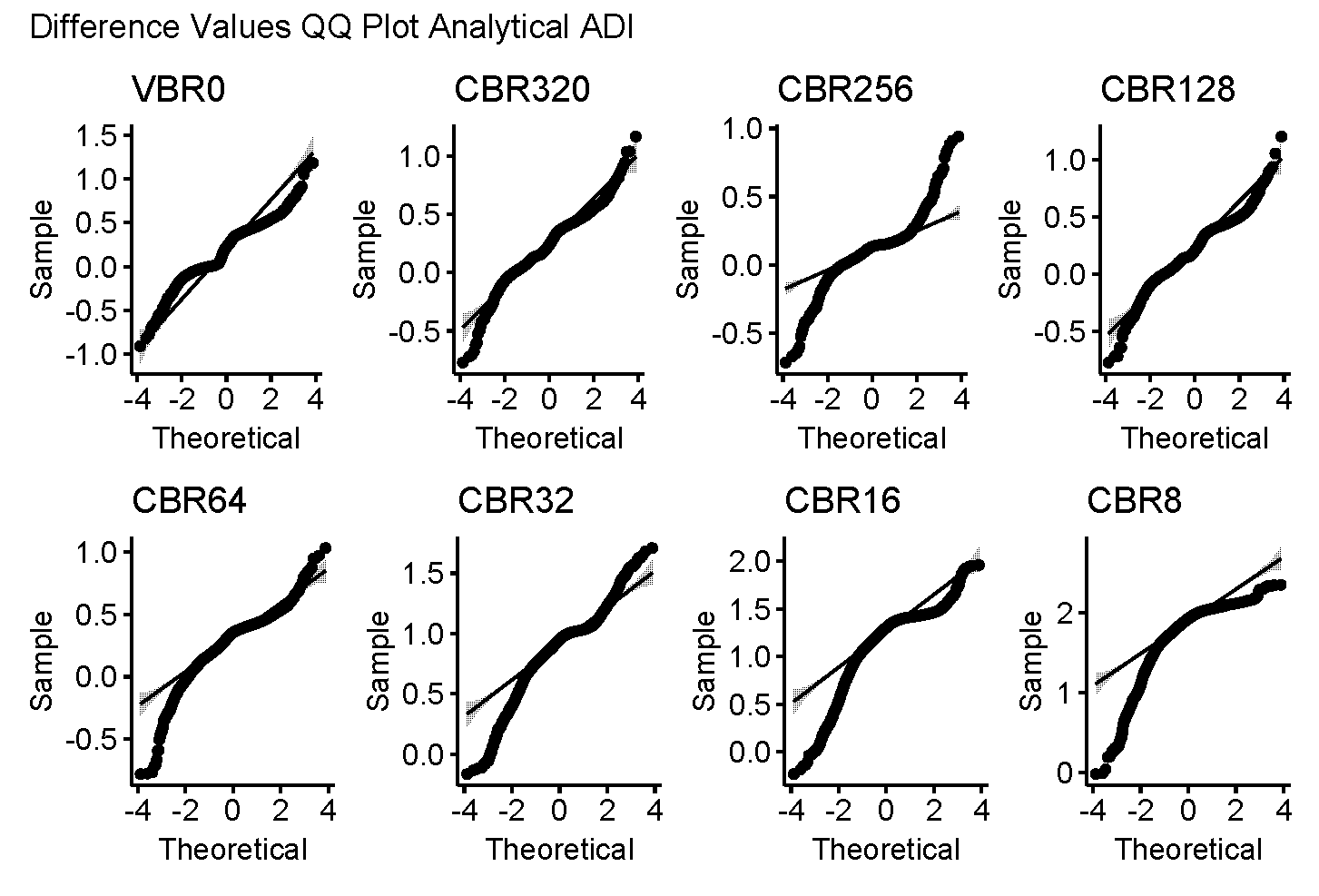

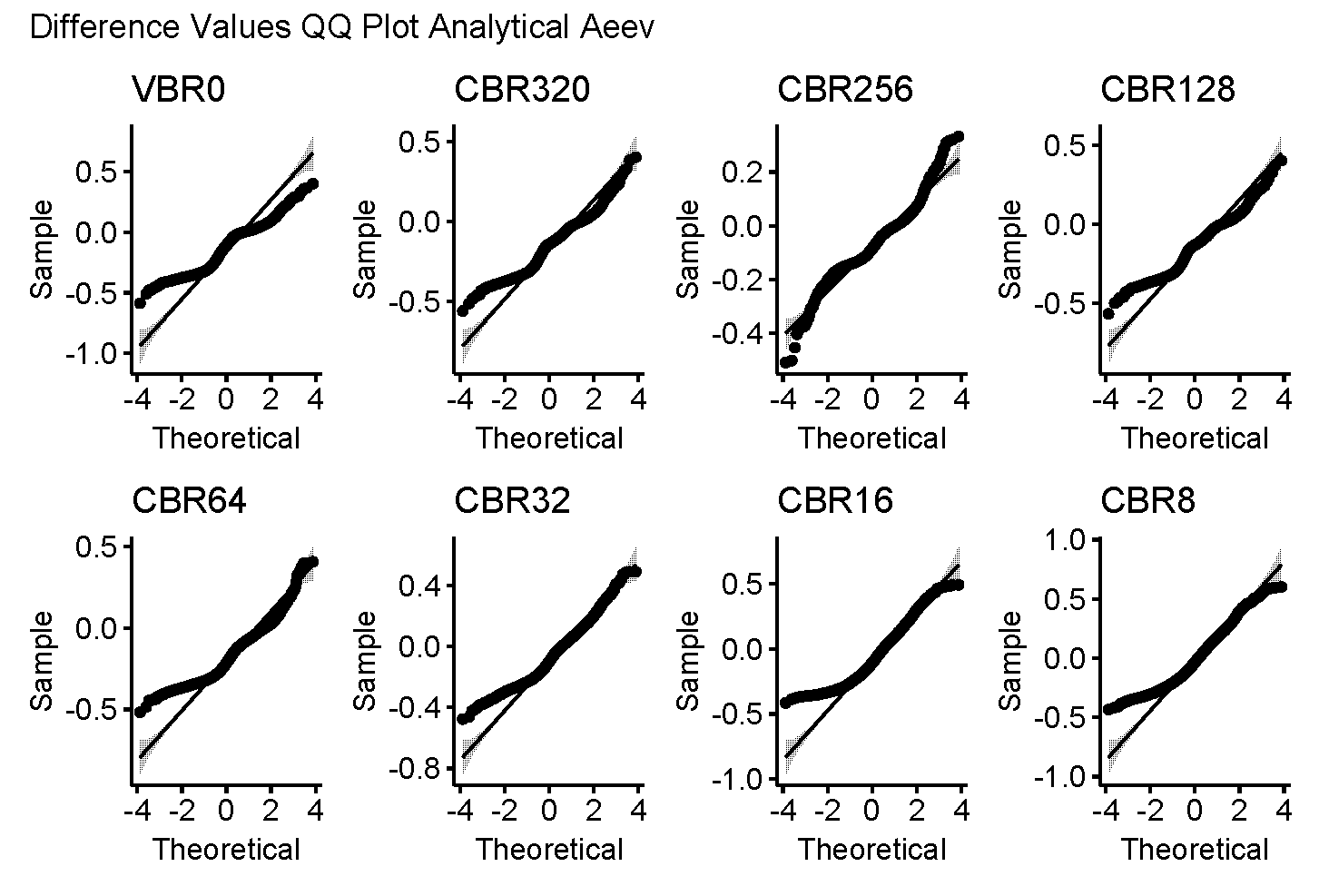

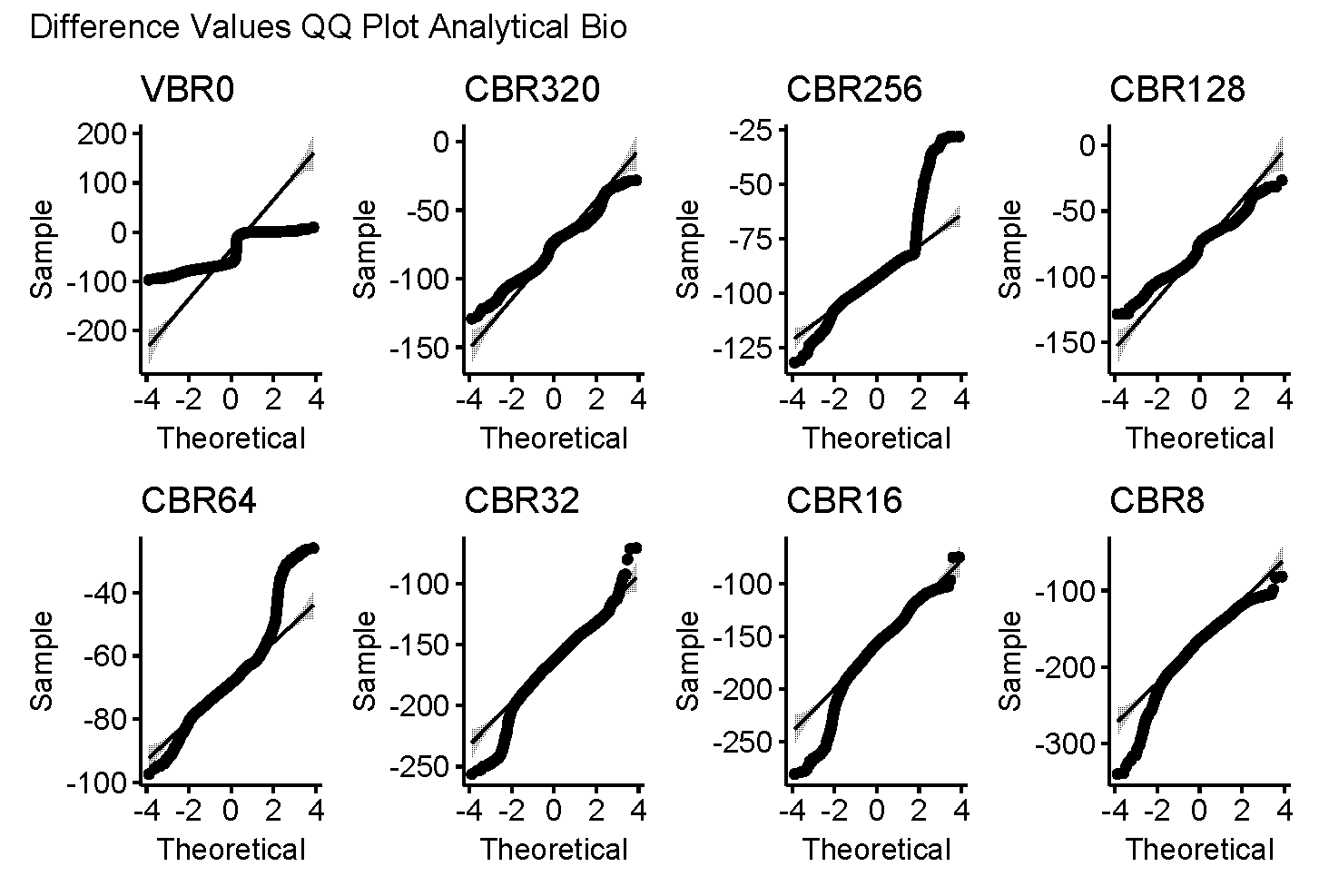

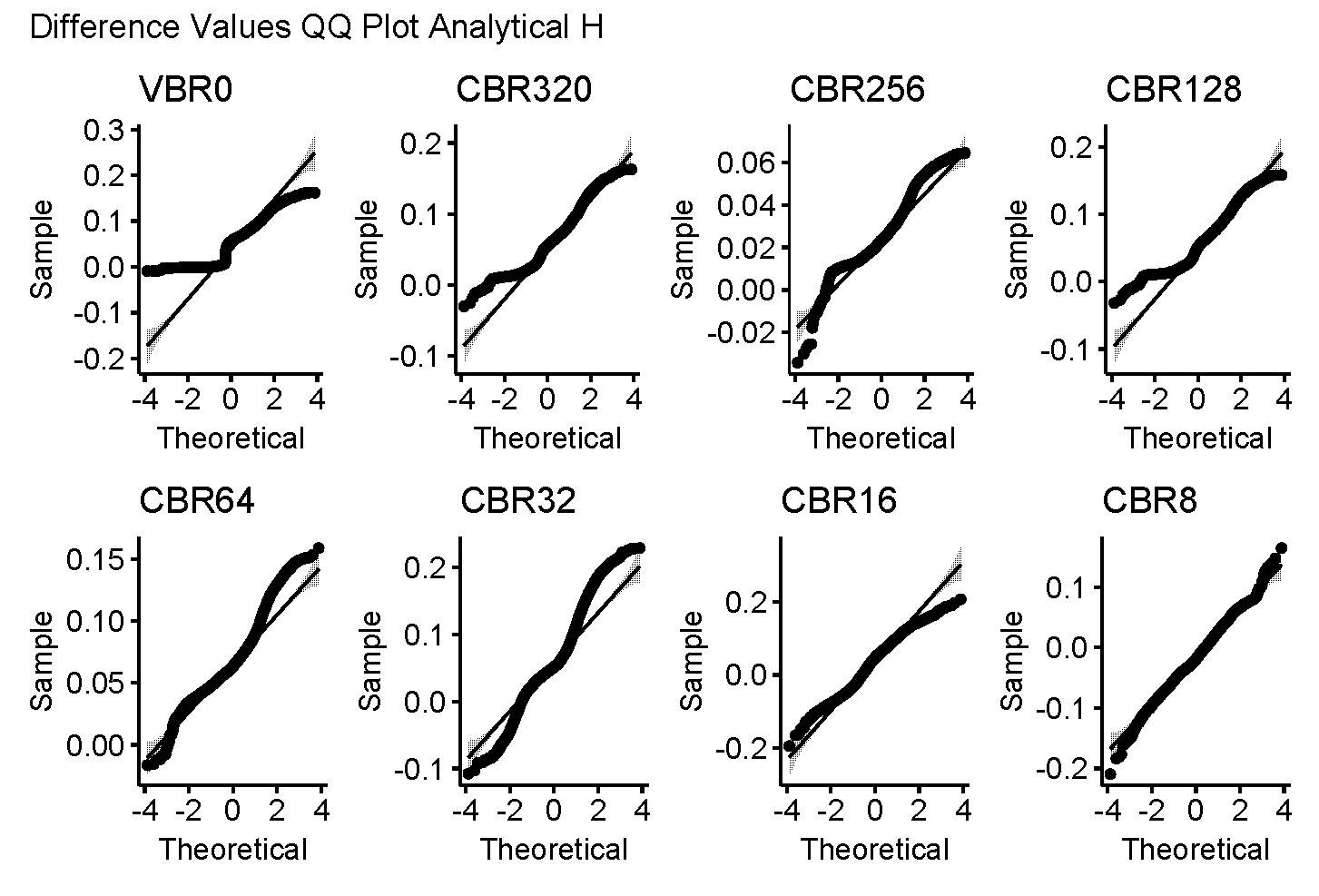

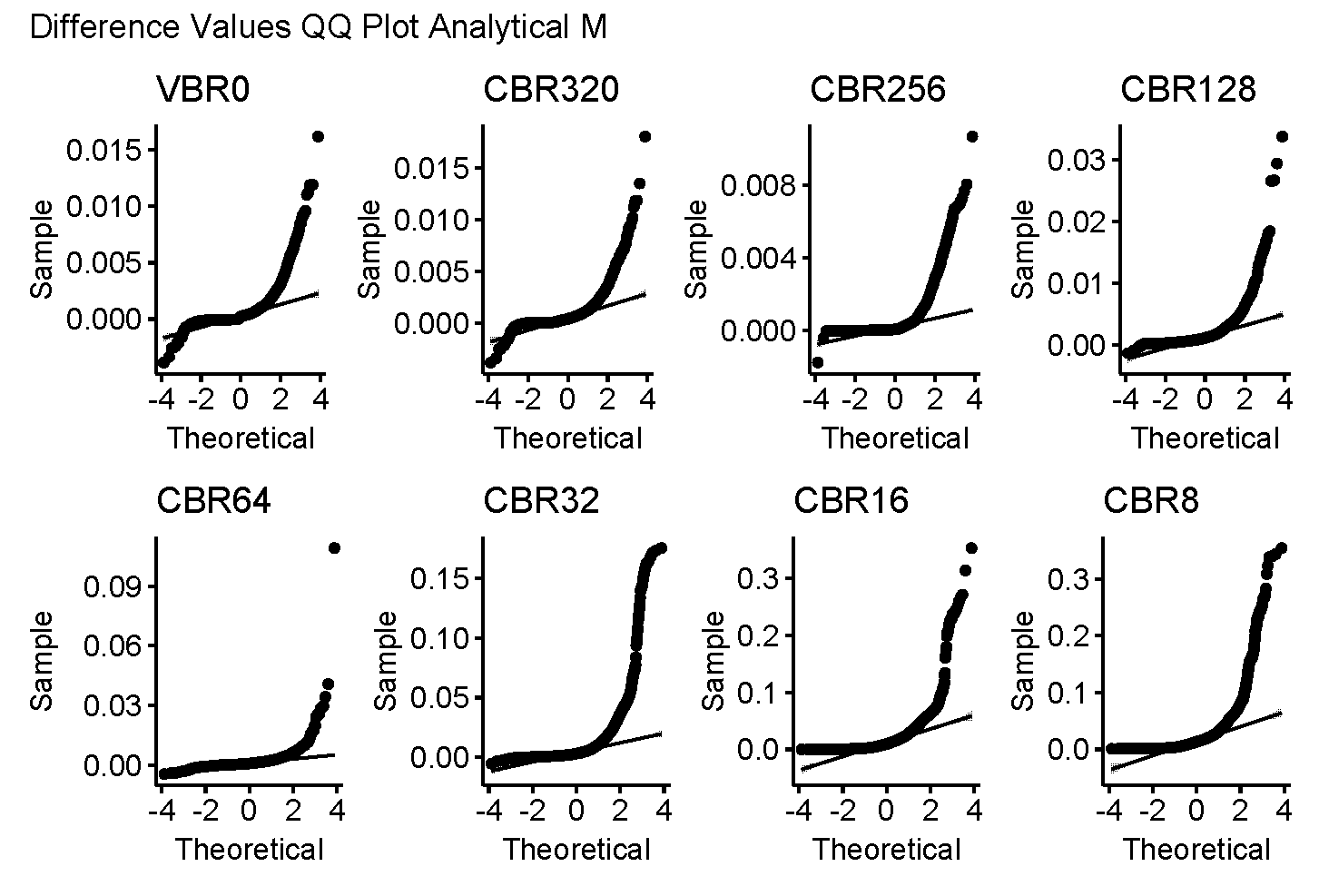

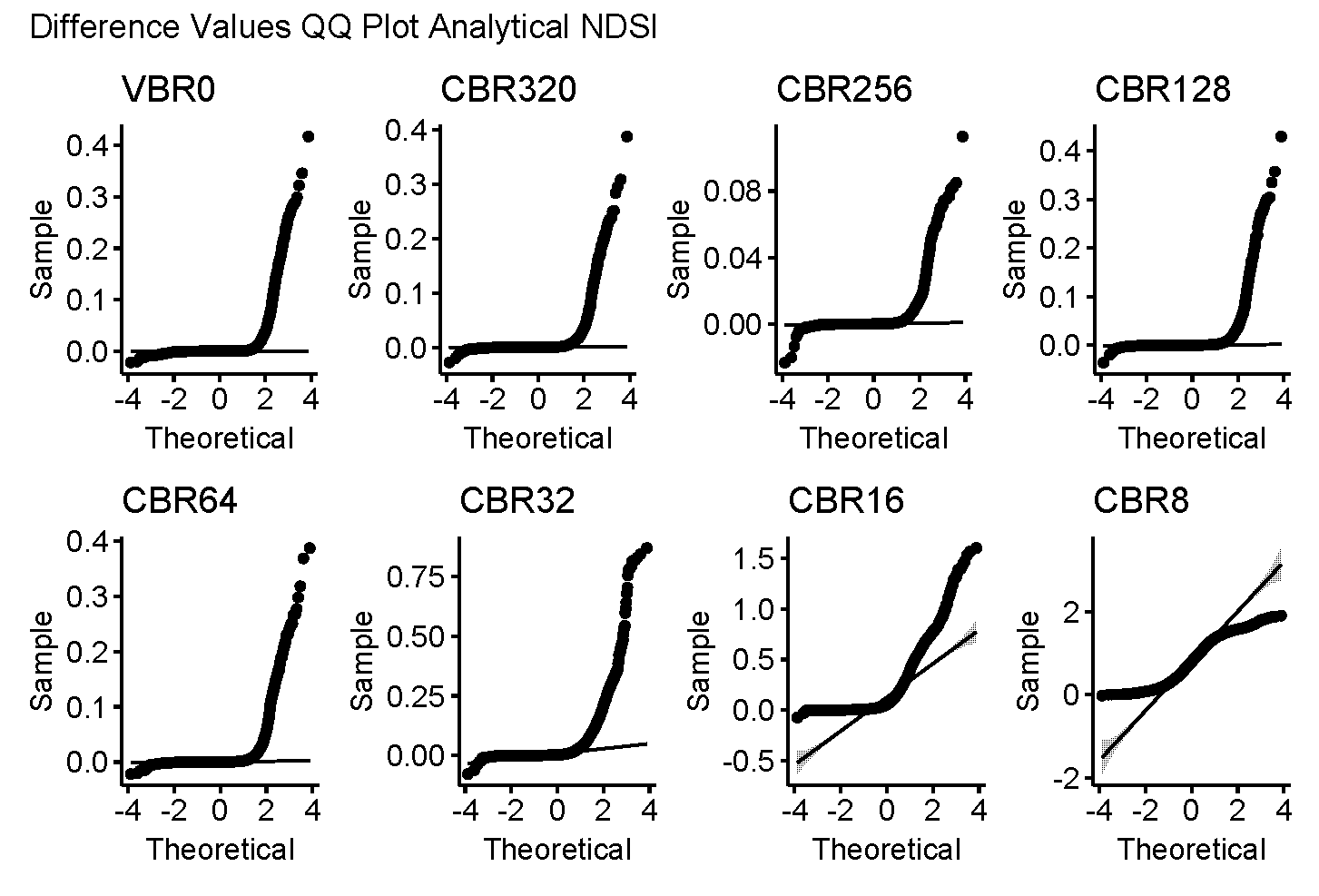


5b – Final Figure Alternatives

**2.5 Minutes: 5 Minutes:**


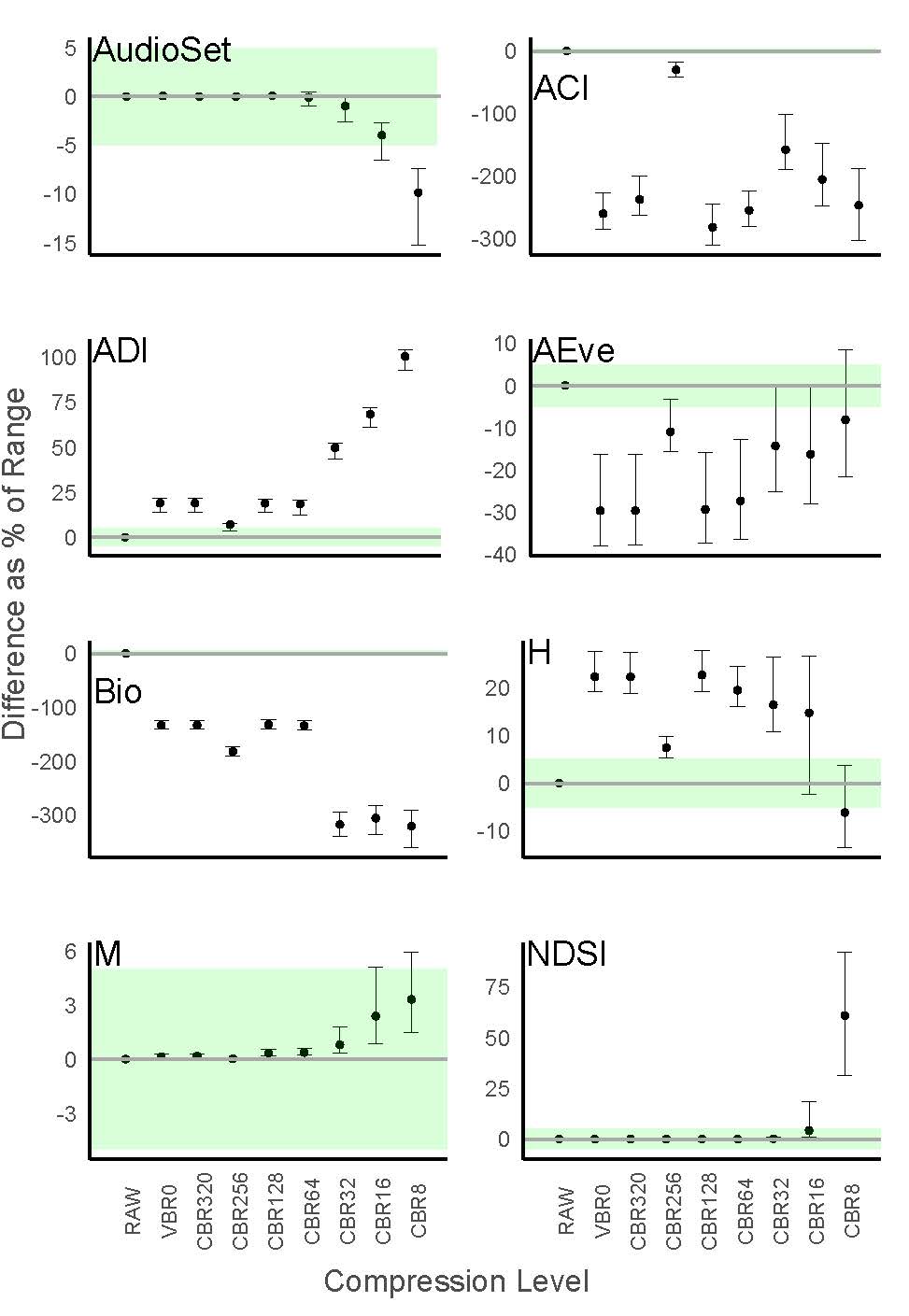

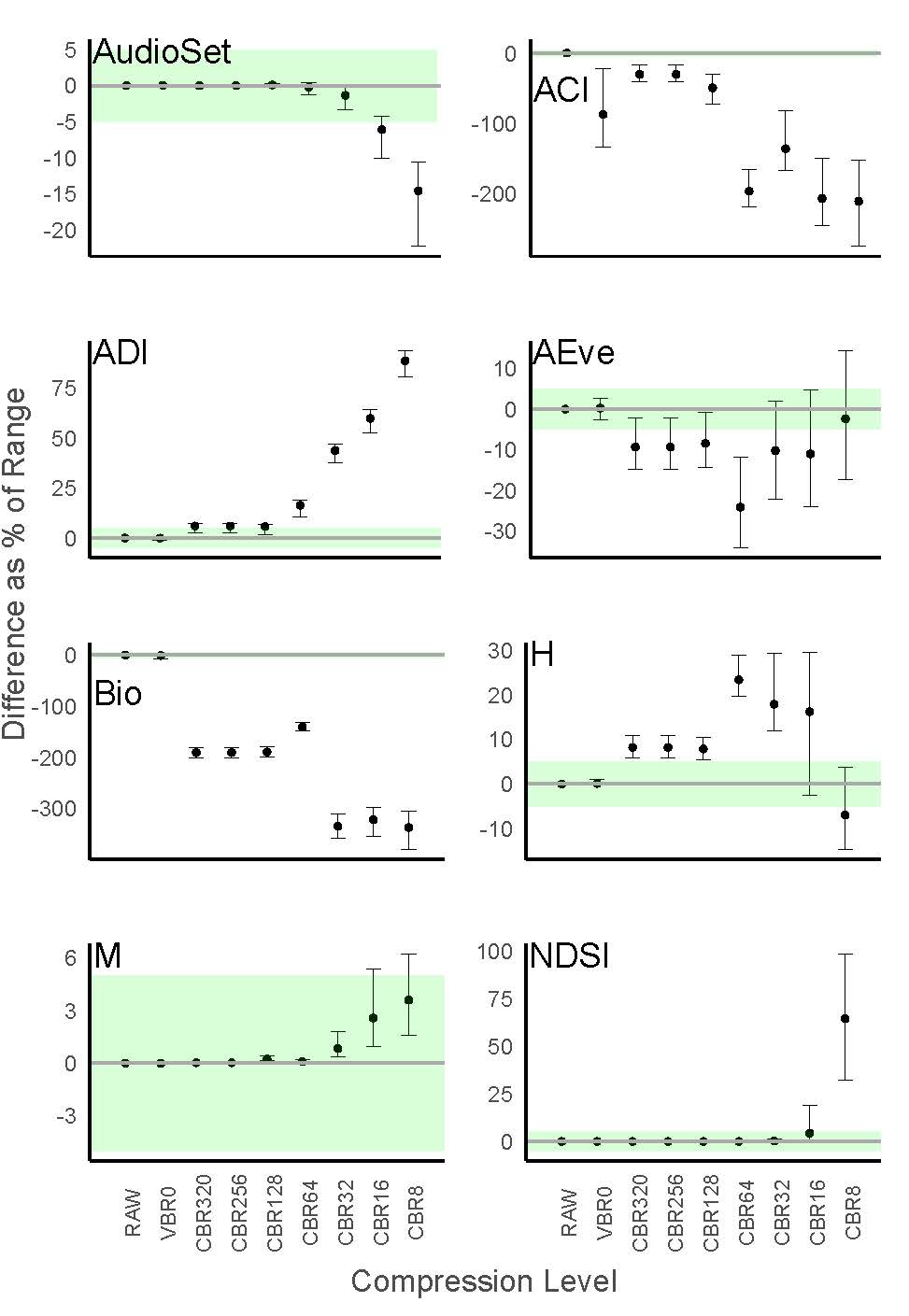


**10 Minutes: 20 Minutes:**


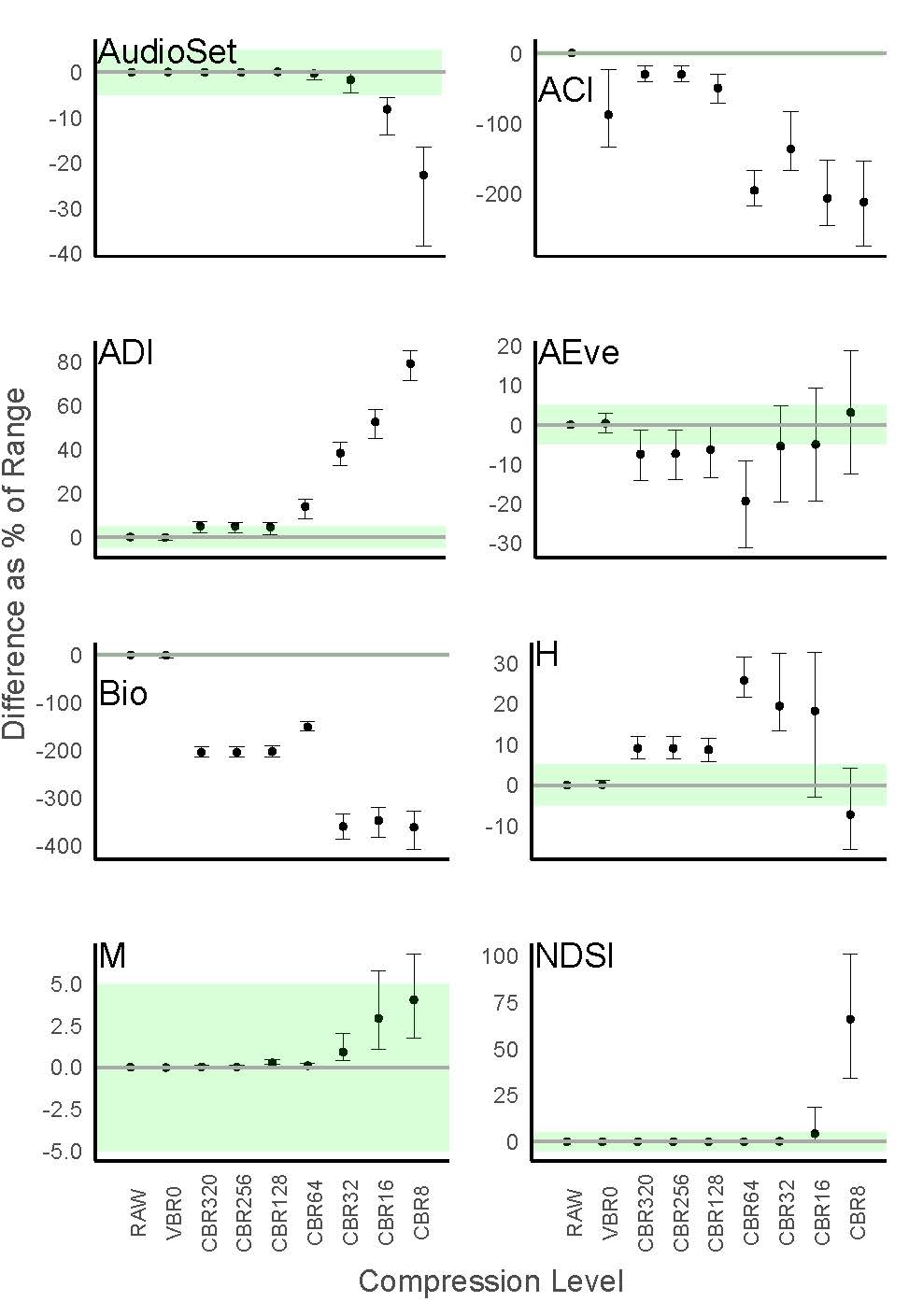

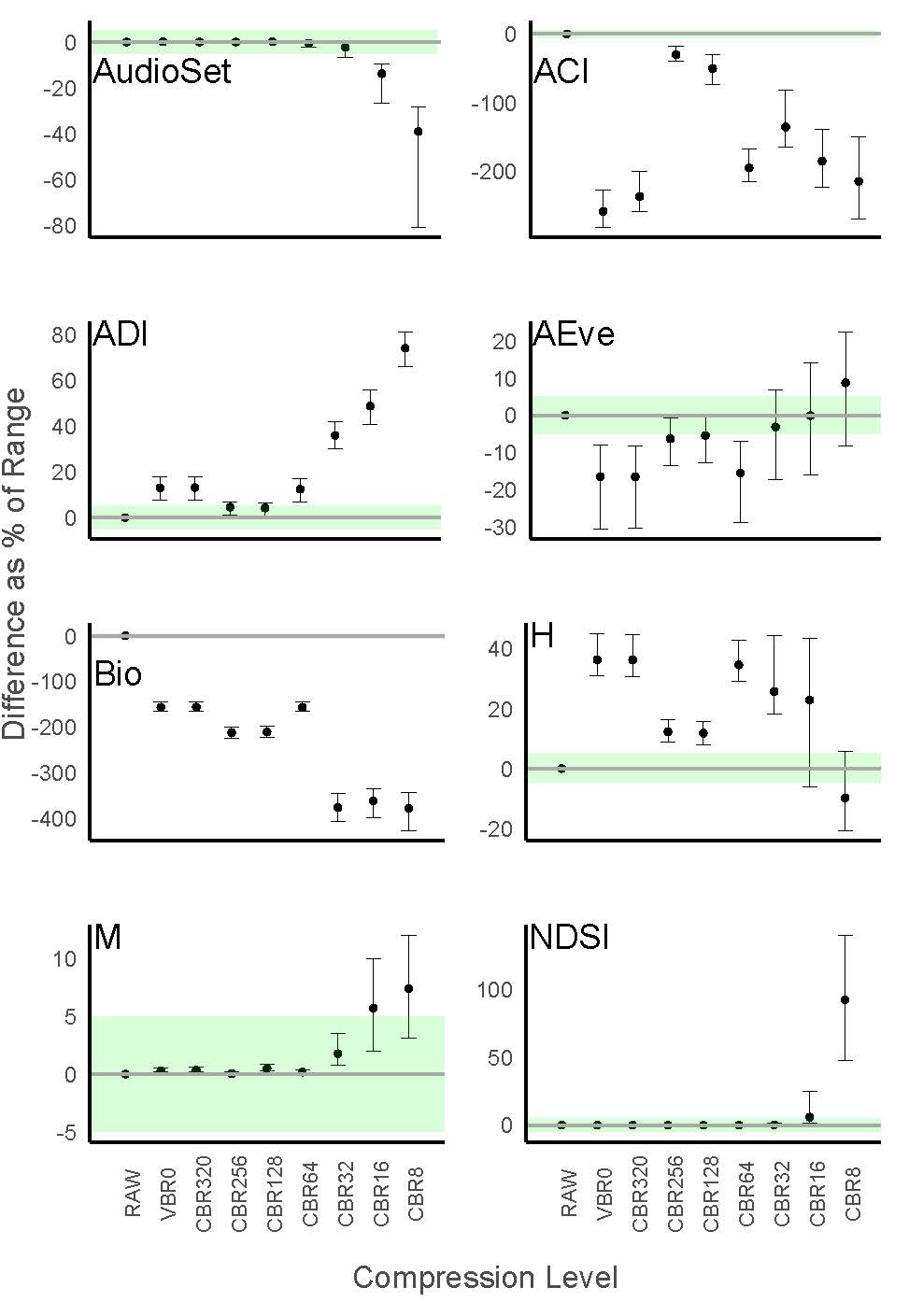


5c – Model Outputs (Spearman’s Rank Correlation Rho)

| **Index** | **Frame Size** | **p-value** | **S** | **rho** | **Alternative hypothesis: Rho is not equal to 0** |
| --- | --- | --- | --- | --- | --- |
| ACI | All | < 2.2e-16 | 5.9908e+13 | 0.4528838 | True |
| ADI | All | < 2.2e-16 | 2.0076e+1 | -0.8334471 | True |
| AEve | All | < 2.2e-16 | 1.0431e+14 | 0.04732955 | True |
| Bio | All | < 2.2e-16 | 2.7865e+13 | 0.7455166 | True |
| H | All | < 2.2e-16 | 1.1409e+14 | -0.04198094 | True |
| M | All | < 2.2e-16 | 1.9495e+14 | -0.7803825 | True |
| NDSI | All | < 2.2e-16 | 1.8306e+14 | -0.6718236 | True |
| AudioSet (+/-) | All | < 2.2e-16 | 1.4664e+14 | -0.3216693 | True |
| AudioSet (absolute) | All | < 2.2e-16 | 2.1968e+14 | -0.9800273 | True |
| ACI | 20min | < 2.2e-16 | 2.3062e+10 | 0.2897066 | True |
| ADI | 20min | < 2.2e-16 | 5.7586e+10 | -0.7736128 | True |
| AEve | 20min | < 2.2e-16 | 3.7679e+10 | -0.1604924 | True |
| Bio | 20min | < 2.2e-16 | 6581545650 | 0.7972928 | True |
| H | 20min | 0.0001731 | 3.0867e+10 | 0.04930388 | True |
| M | 20min | < 2.2e-16 | 5.678e+10 | -0.7487885 | True |
| NDSI | 20min | < 2.2e-16 | 5.4404e+10 | -0.6756143 | True |
| AudioSet (+/-) | 20min | < 2.2e-16 | 4.3576e+10 | -0.324907 | True |
| AudioSet (absolute) | 20min | < 2.2e-16 | 6.5184e+10 | -0.9818801 | True |
| ACI | 10min | < 2.2e-16 | 7.7613e+10 | 0.6993322 | True |
| ADI | 10min | < 2.2e-16 | 4.6993e+11 | -0.820476 | True |
| AEve | 10min | 7.569e-05 | 2.4864e+11 | 0.03678488 | True |
| Bio | 10min | < 2.2e-16 | 7.1987e+10 | 0.7211259 | True |
| H | 10min | < 2.2e-16 | 3.011e+11 | -0.1664376 | True |
| M | 10min | < 2.2e-16 | 4.5137e+11 | -0.7485807 | True |
| NDSI | 10min | < 2.2e-16 | 4.3252e+11 | -0.6755327 | True |
| AudioSet (+/-) | 10min | < 2.2e-16 | 3.4289e+11 | -0.3224749 | True |
| AudioSet (absolute) | 10min | < 2.2e-16 | 5.1372e+11 | -0.9813791 | True |
| ACI | 5min | < 2.2e-16 | 6.2663e+11 | 0.6977385 | True |
| ADI | 5min | < 2.2e-16 | 3.8019e+12 | -0.8339001 | True |
| AEve | 5min | < 2.2e-16 | 1.7817e+12 | 0.1405795 | True |
| Bio | 5min | < 2.2e-16 | 5.7816e+11 | 0.7211196 | True |
| H | 5min | < 2.2e-16 | 2.4202e+12 | -0.1674052 | True |
| M | 5min | < 2.2e-16 | 3.6248e+12 | -0.7484442 | True |
| NDSI | 5min | < 2.2e-16 | 3.4694e+12 | -0.6734986 | True |
| AudioSet (+/-) | 5min | < 2.2e-16 | 2.7754e+12 | -0.3239534 | True |
| AudioSet (absolute) | 5min | < 2.2e-16 | 4.1512e+12 | -0.9802393 | True |
| ACI | 2.5min | < 2.2e-16 | 1.1169e+13 | 0.3289954 | True |
| ADI | 2.5min | < 2.2e-16 | 3.0666e+13 | -0.8423228 | True |
| AEve | 2.5min | 0.00296 | 1.6416e+13 | 0.01379665 | True |
| Bio | 2.5min | < 2.2e-16 | 4.0057e+12 | 0.7593467 | True |
| H | 2.5min | < 2.2e-16 | 1.5846e+13 | 0.04798813 | True |
| M | 2.5min | < 2.2e-16 | 3.0169e+13 | -0.8124801 | True |
| NDSI | 2.5min | < 2.2e-16 | 2.7774e+13 | -0.6686069 | True |
| AudioSet (+/-) | 2.5min | < 2.2e-16 | 2.2333e+13 | -0.3199297 | True |
| AudioSet (absolute) | 2.5min | < 2.2e-16 | 3.3499e+13 | -0.979805 | True |

**6 – Impact of Recording Schedule: Recording Length on Variance**

6a Levene’s Test for Homogeneity of Variance

| index | P value |  | Index | P value |  | Index | P value |
| --- | --- | --- | --- | --- | --- | --- | --- |
| ACI | 0.841637 |  | feat42 | 0.706519 |  | feat90 | 0.15218 |
| ADI | 1.78E-39 |  | feat43 | 2.68E-05 |  | feat91 | 0.932792 |
| Aeev | 0.028428 |  | feat44 | 3.54E-07 |  | feat92 | 1.96E-15 |
| Bio | 0.794537 |  | feat45 | 0.779526 |  | feat93 | 0.906691 |
| H | 0.004092 |  | feat46 | 0.892285 |  | feat94 | 1.01E-08 |
| M | 0.067758 |  | feat47 | 0.000662 |  | feat95 | 0.86466 |
| NDSI | 0.714233 |  | feat48 | 1.05E-09 |  | feat96 | 2.87E-09 |
| feat1 | 0.837382 |  | feat49 | 0.000274 |  | feat97 | 0.824457 |
| feat2 | 0.959618 |  | feat50 | 9.91E-06 |  | feat98 | 0.979221 |
| feat3 | 1.58E-06 |  | feat51 | 0.999517 |  | feat99 | 0.797975 |
| feat4 | 0.988474 |  | feat52 | 0.002494 |  | feat100 | 0.985947 |
| feat5 | 0.981484 |  | feat53 | 0.723206 |  | feat101 | 0.998016 |
| feat6 | 0.912722 |  | feat54 | 0.820143 |  | feat102 | 1.46E-07 |
| feat7 | 0.980983 |  | feat55 | 0.462347 |  | feat103 | 2.92E-05 |
| feat8 | 0.99822 |  | feat56 | 2.27E-06 |  | feat104 | 0.093257 |
| feat9 | 0.800522 |  | feat57 | 4.58E-13 |  | feat105 | 0.997949 |
| feat10 | 0.119387 |  | feat58 | 0.99986 |  | feat106 | 0.999947 |
| feat11 | 0.999692 |  | feat59 | 0.987711 |  | feat107 | 0.925252 |
| feat12 | 0.901465 |  | feat60 | 0.005519 |  | feat108 | 0.997711 |
| feat13 | 0.989909 |  | feat61 | 0.522512 |  | feat109 | 0.334764 |
| feat14 | 0.000972 |  | feat62 | 0.968115 |  | feat110 | 6.68E-05 |
| feat15 | 8.75E-06 |  | feat63 | 0.970316 |  | feat111 | 0.998584 |
| feat16 | 0.781888 |  | feat64 | 0.996904 |  | feat112 | 1.28E-08 |
| feat17 | 2.41E-14 |  | feat65 | 0.001259 |  | feat113 | 7.91E-14 |
| feat18 | 0.407886 |  | feat66 | 0.002933 |  | feat114 | 0.284966 |
| feat19 | 0.955162 |  | feat67 | 0.73536 |  | feat115 | 0.974201 |
| feat20 | 0.406722 |  | feat68 | 0.000104 |  | feat116 | 0.99686 |
| feat21 | 4.56E-11 |  | feat69 | 0.999244 |  | feat117 | 0.999671 |
| feat22 | 0.092009 |  | feat70 | 0.993015 |  | feat118 | 0.519933 |
| feat23 | 0.99988 |  | feat71 | 0.934927 |  | feat119 | 0.304281 |
| feat24 | 0.01768 |  | feat72 | 0.03893 |  | feat120 | 5.41E-11 |
| feat25 | 2.69E-12 |  | feat73 | 0.930866 |  | feat121 | 0.050387 |
| feat26 | 0.996596 |  | feat74 | 0.972913 |  | feat122 | 0.983873 |
| feat27 | 0.001689 |  | feat75 | 0.563142 |  | feat123 | 0.000172 |
| feat28 | 0.185621 |  | feat76 | 0.000475 |  | feat124 | 8.04E-10 |
| feat29 | 0.50037 |  | feat77 | 7.73E-05 |  | feat125 | 0.693315 |
| feat30 | 0.410133 |  | feat78 | 1.63E-08 |  | feat126 | 0.994528 |
| feat31 | 0.255612 |  | feat79 | 3.75E-14 |  | feat127 | 0.004084 |
| feat32 | 0.974623 |  | feat80 | 0.526298 |  | feat128 | 0.553217 |
| feat33 | 0.01168 |  | feat81 | 0.924814 |  |  |  |
| feat34 | 2.89E-10 |  | feat82 | 0.800698 |  |  |  |
| feat35 | 0.867947 |  | feat83 | 0.939481 |  |  |  |
| feat36 | 3.25E-09 |  | feat84 | 0.320144 |  |  |  |
| feat37 | 0.995959 |  | feat85 | 0.289219 |  |  |  |
| feat38 | 6.07E-06 |  | feat86 | 4.56E-08 |  |  |  |
| feat39 | 0.092141 |  | feat87 | 0.998176 |  |  |  |
| feat40 | 3.58E-05 |  | feat88 | 3.07E-05 |  |  |  |
| feat41 | 5.58E-11 |  | feat89 | 0.981046 |  |  |  |

**Levene’s Test for Homogeneity of Variance**

Analytical Indices: 3/7 indices have a p<0.05 meaning variances are not equal

AudioSet Indices: 46/128 indices have a p<0.05 meaning variances are not equal

**7 – Impact of Index Type: Confusion Matrices (Figure 2)**

7a – Alternative Matrices

| Analytical | **20min** |  |  |  | AudioSet |  |  |  |
| --- | --- | --- | --- | --- | --- | --- | --- | --- |
| Grassland | 124 | 22 | 6 |  | Grassland | 147 | 4 | 1 |
| Secondary | 29 | 99 | 11 |  | Secondary | 2 | 126 | 13 |
| Primary | 5 | 11 | 123 |  | Primary | 2 | 4 | 133 |
|  |  |  |  |  |  |  |  |  |
| Analytical | **10min** |  |  |  | AudioSet |  |  |  |
| Grassland | 235 | 49 | 13 |  | Grassland | 292 | 6 | 2 |
| Secondary | 46 | 213 | 20 |  | Secondary | 3 | 253 | 26 |
| Primary | 7 | 23 | 248 |  | Primary | 6 | 8 | 264 |
|  |  |  |  |  |  |  |  |  |
| Analytical | **5min** |  |  |  | AudioSet |  |  |  |
| Grassland | 484 | 67 | 49 |  | Grassland | 585 | 9 | 11 |
| Secondary | 97 | 421 | 46 |  | Secondary | 11 | 508 | 44 |
| Primary | 9 | 61 | 486 |  | Primary | 17 | 14 | 521 |
|  |  |  |  |  |  |  |  |  |
| Analytical | **2.5min** |  |  |  | AudioSet |  |  |  |
| Grassland | 1011 | 126 | 75 |  | Grassland | 1160 | 24 | 32 |
| Secondary | 193 | 837 | 97 |  | Secondary | 28 | 996 | 104 |
| Primary | 13 | 138 | 957 |  | Primary | 33 | 39 | 1040 |

7b – Accuracy, Precision and Recall Statistics

| **Index Type** | **Frame Size** | **Accuracy** | **Precision** | **Recall** |
| --- | --- | --- | --- | --- |
| Analytical | 20Min | 80.47 | 80.45 | 80.43 |
| Analytical | 10Min | 81.50 | 81.53 | 81.56 |
| Analytical | 5Min | 80.87 | 80.79 | 80.91 |
| Analytical | 2.5Min | 81.38 | 81.28 | 81.35 |
| AudioSet | 20Min | 93.98 | 93.95 | 93.92 |
| AudioSet | 10Min | 94.06 | 94.06 | 94.00 |
| AudioSet | 5Min | 93.84 | 93.85 | 93.77 |
| AudioSet | 2.5min | 92.48 | 93.85 | 93.92 |

**8 – Impact of Temporal Splitting (Figure 4)**

8a – Figure Alternatives

**2.5 Minute:**


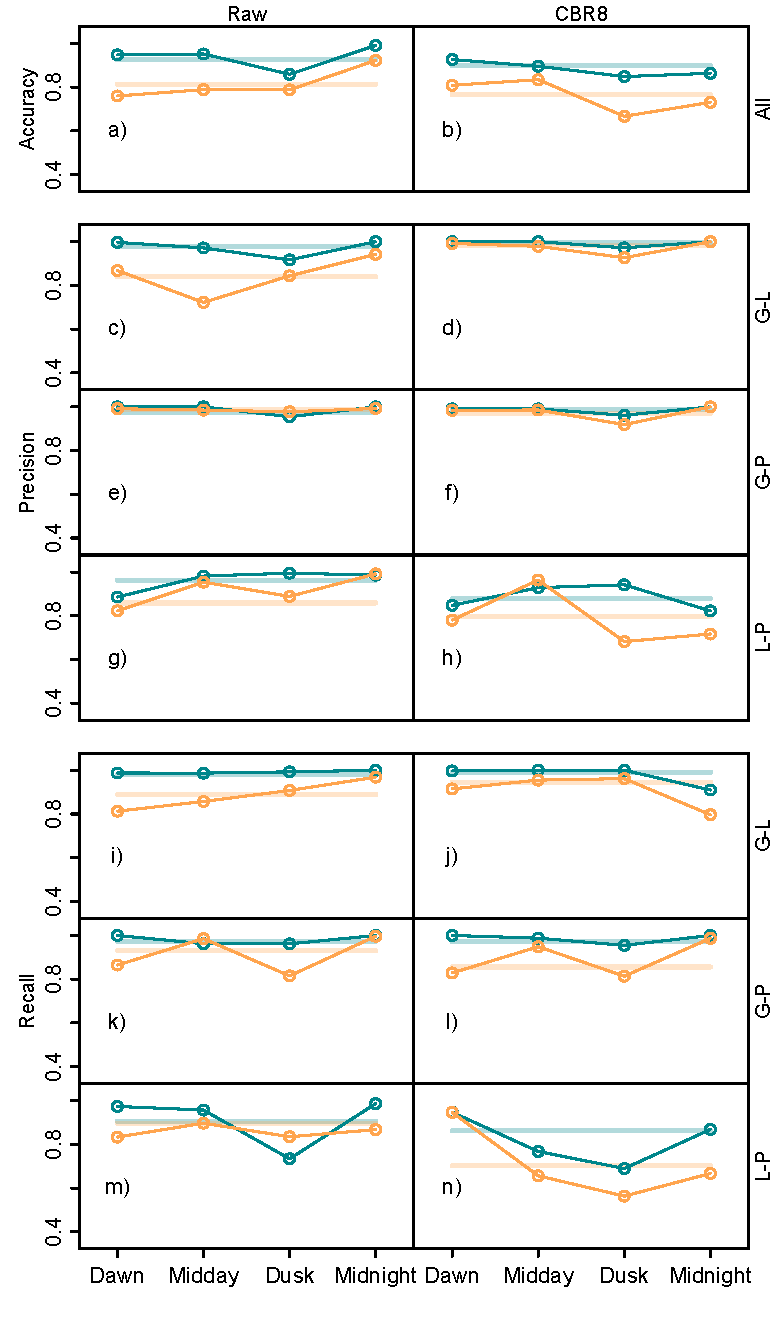


**5 Minute:**


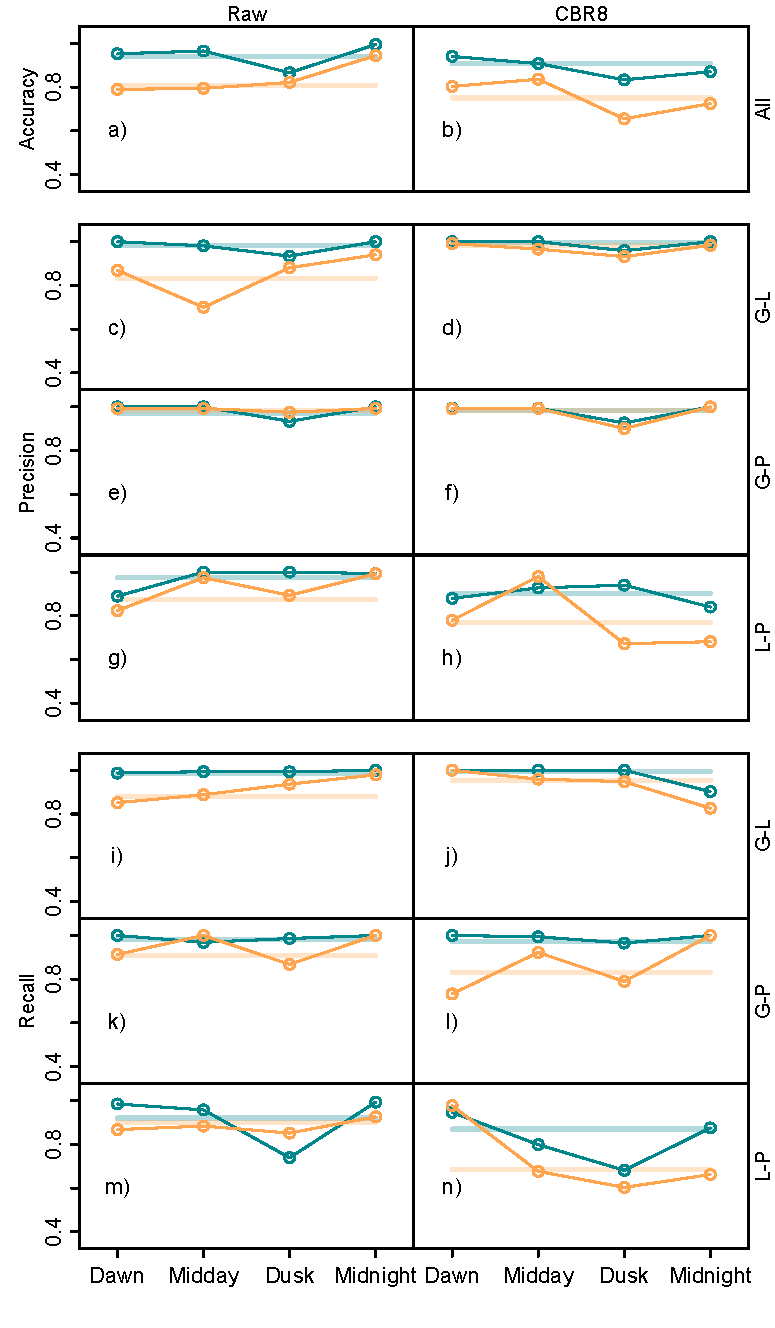


**10 Minute**


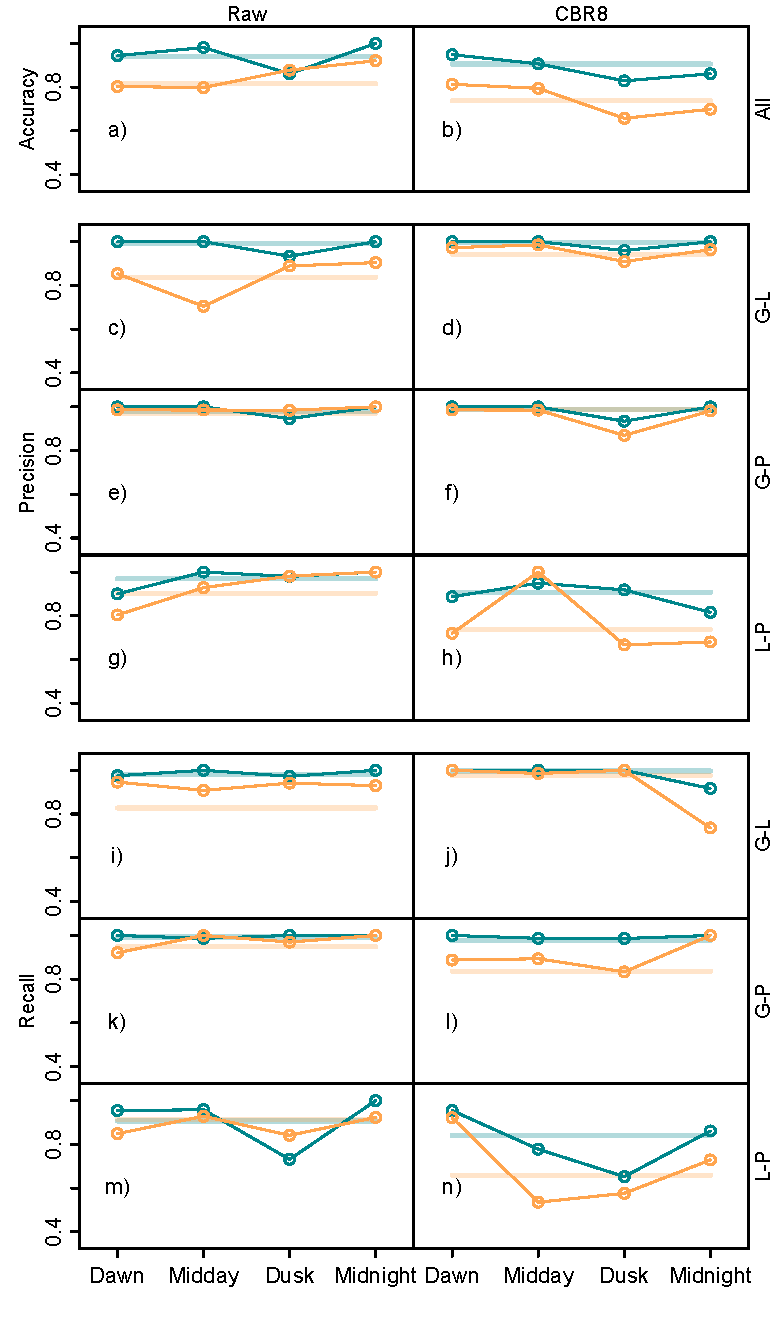


**20 Minute**


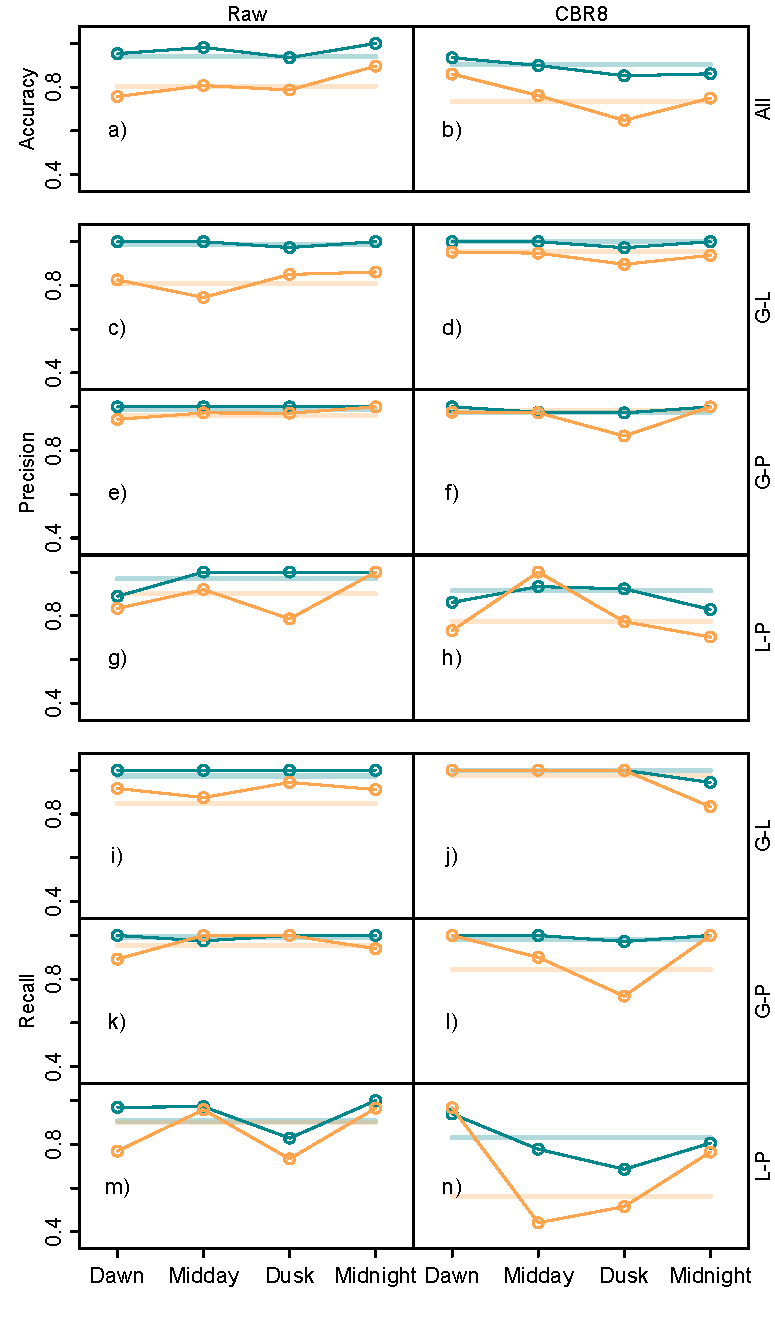


**9 – Synthesis: Beta Regression Modelling the Contribution of all Parameters**

9a – Data Variance (ϕ component)


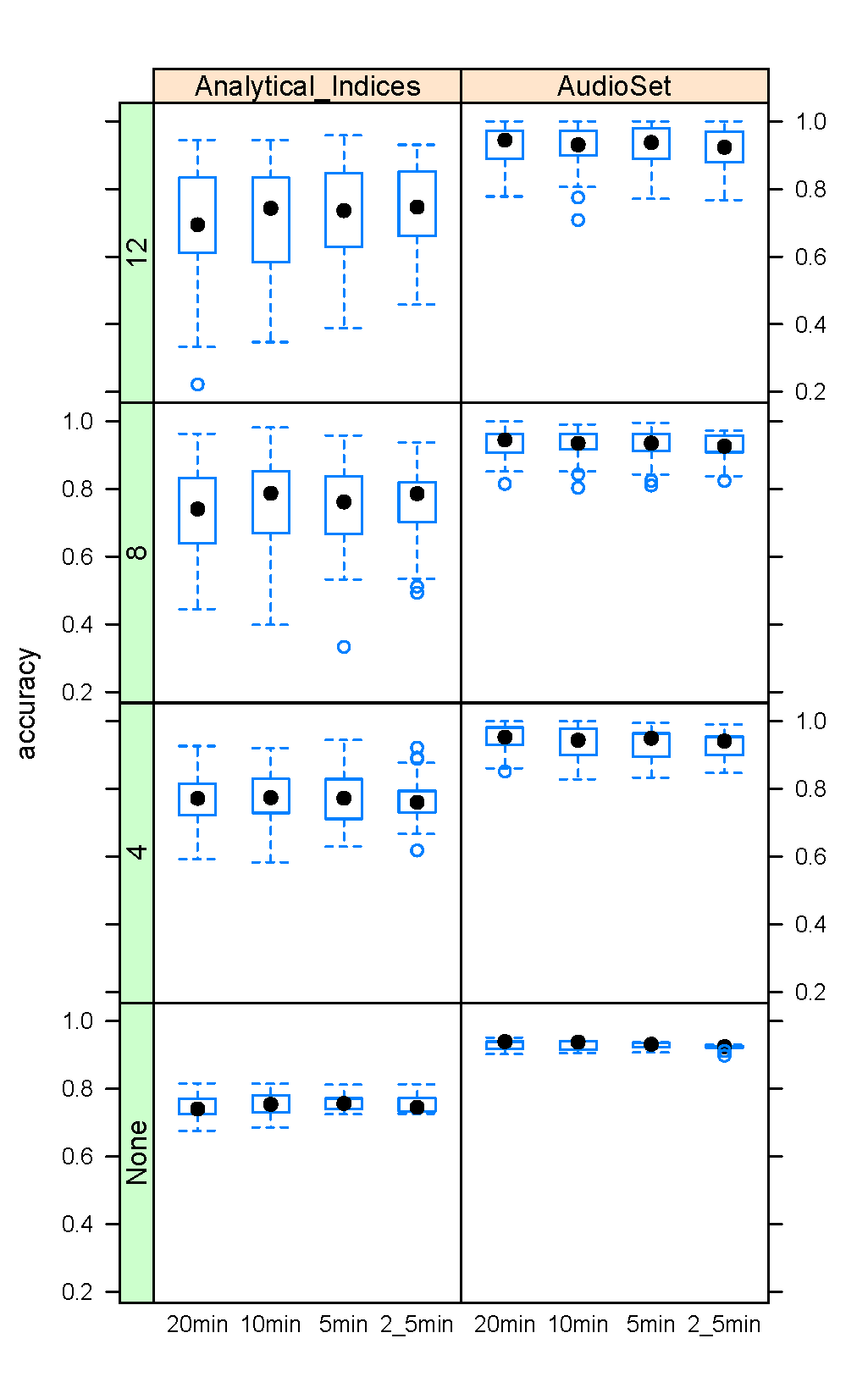


9b – AIC values of models

| Model | Df | AIC |
| --- | --- | --- |
| Linear Model | 47 | -3428.738 |
| Beta Regression | 47 | -4368.424 |
| **Beta Regression + ϕ** | **54** | **-4682.399** |
| Beta Regression + ϕ (continuous temporal splits + frame size) | 18 | -4516.453 |

Maximal Model: accuracy_t ~ (log10(file.size) + chunks + frame.size) ^ 2 * index.type

9c Precision and Recall Figure Outputs


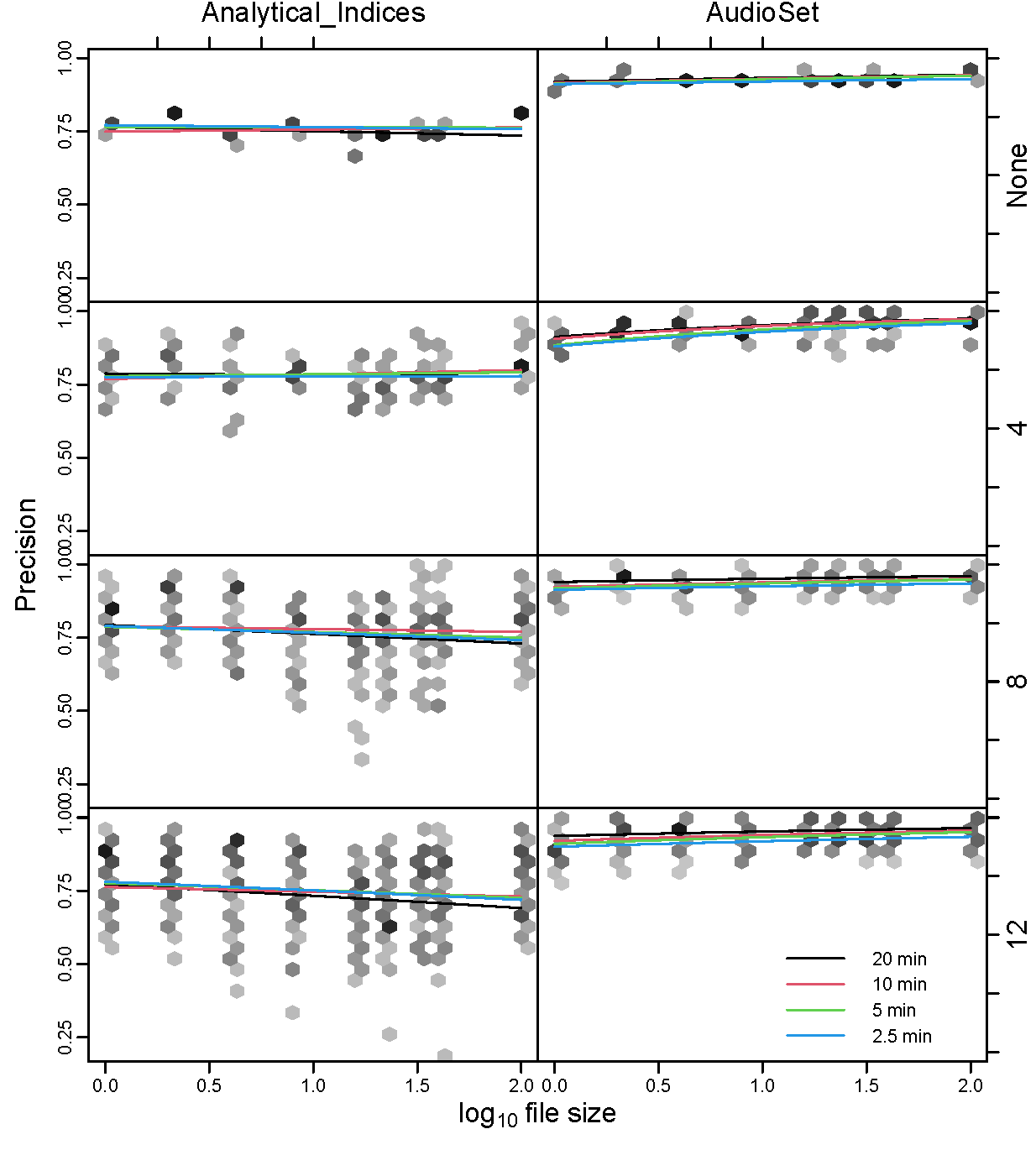


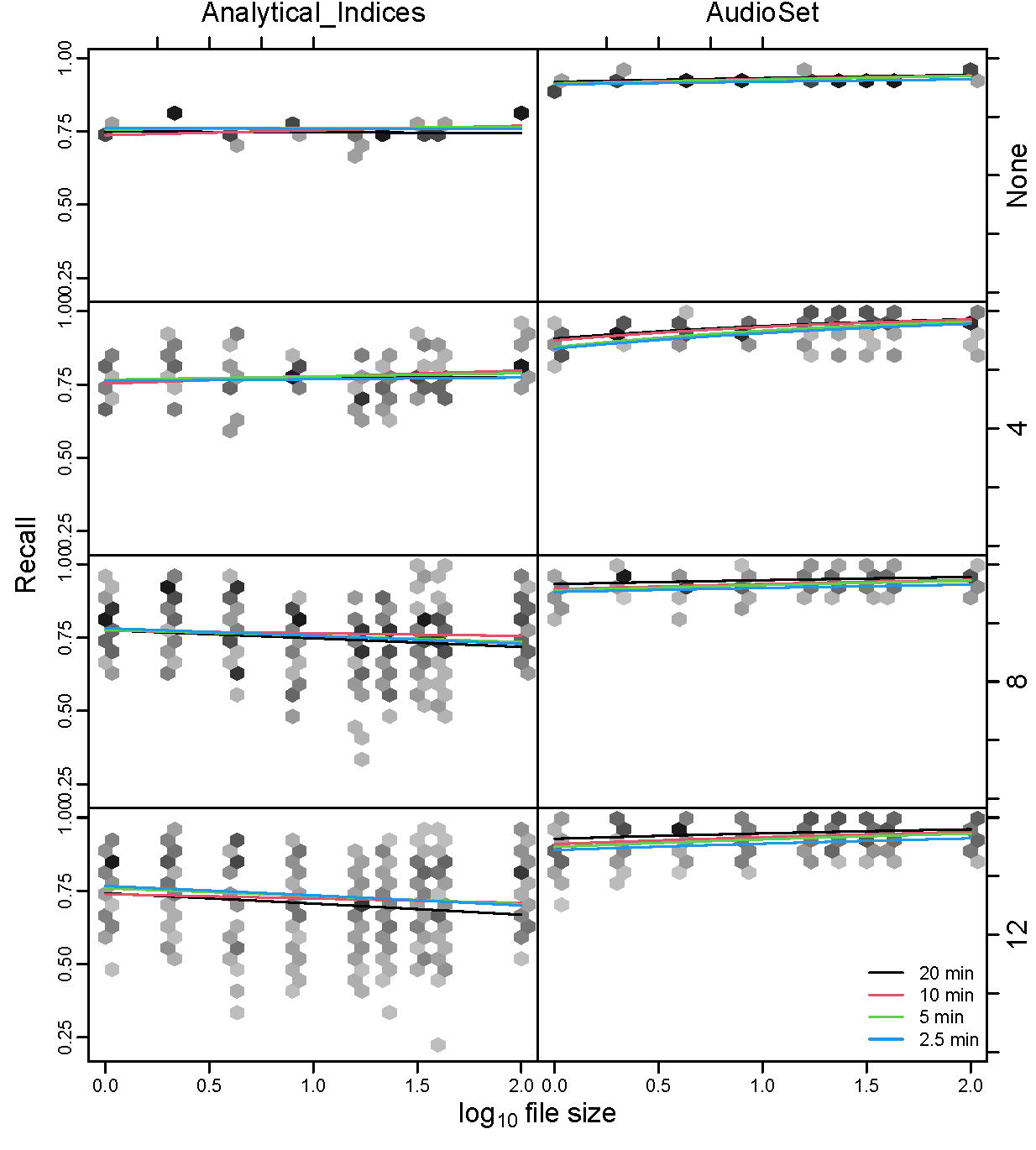


9d – Beta Regression Output Statistics

**ACCURACY:**

Linear model AOV:

|  | df | Sum Sq | Mean Sq | F Value | Pr(>F) |
| --- | --- | --- | --- | --- | --- |
| log10(file.size) | 1 | 0.000 | 0.000 | 0.000 | 0.99740 |
| chunks | 3 | 0.243 | 0.081 | 9.644 | 2.59e-06 *** |
| frame.size | 3 | 0.007 | 0.002 | 0.271 | 0.84646 |
| index.type | 1 | 15.980 | 15.980 | 1899.119 | < 2e-16 *** |
| log10(file.size):chunks | 3 | 0.049 | 0.016 | 1.947 | 0.12002 |
| log10(file.size):frame.size | 3 | 0.008 | 0.003 | 0.301 | 0.82446 |
| chunks:frame.size | 9 | 0.016 | 0.002 | 0.215 | 0.99238 |
| log10(file.size):index.type | 1 | 0.288 | 0.288 | 34.231 | 5.83e-09 *** |
| chunks:index.type | 3 | 0.119 | 0.040 | 4.726 | 0.00274 ** |
| frame.size:index.type | 3 | 0.076 | 0.025 | 3.009 | 0.02920 * |
| log10(file.size):chunks:index.type | 3 | 0.040 | 0.013 | 1.599 | 0.18771 |
| log10(file.size):frame.size:index.type | 3 | 0.016 | 0.005 | 0.634 | 0.59286 |
| chunks:frame.size:index.type | 9 | 0.009 | 0.001 | 0.121 | 0.99919 |
| Residuals | 1753 | 14.751 | 0.008 |  |  |

Signif. codes: 0 ‘***’ 0.001 ‘**’ 0.01 ‘*’ 0.05 ‘.’ 0.1 ‘ ’ 1

Betareg Anova of the Maximal Model: (accuracy_t ~ (log10(file.size) + chunks + frame.size) ^ 2 * index.type | index.type * chunks)

Analysis of Deviance Table (Type II tests)

|  | Df | Chisq | Pr(>Chisq) |
| --- | --- | --- | --- |
| log10(file.size) | 1 | 26.2128 | 3.058e-07 *** |
| chunks | 3 | 31.6818 | 6.107e-07 *** |
| frame.size | 3 | 15.7820 | 0.0012568 ** |
| index.type | 1 | 2985.9825 | < 2.2e-16 *** |
| log10(file.size):chunks | 3 | 18.0278 | 0.0004341 *** |
| log10(file.size):frame.size | 3 | 2.9280 | 0.4028558 |
| chunks:frame.size | 9 | 6.3156 | 0.7079609 |
| log10(file.size):index.type | 1 | 59.0065 | 1.572e-14 *** |
| chunks:index.type | 3 | 7.1061 | 0.0685927 . |
| frame.size:index.type | 3 | 36.2699 | 6.566e-08 *** |
| log10(file.size):chunks:index.type | 3 | 13.0715 | 0.0044844 ** |
| log10(file.size):frame.size:index.type | 3 | 0.8071 | 0.8477715 |
| chunks:frame.size:index.type | 9 | 7.1524 | 0.6212537 |

Signif. codes: 0 ‘***’ 0.001 ‘**’ 0.01 ‘*’ 0.05 ‘.’ 0.1 ‘ ’ 1

Maxima Model Summary:

**betareg(formula = accuracy_t ~ log10(file.size) + chunks + frame.size + index.type +**

**log10(file.size):chunks + log10(file.size):frame.size + chunks:frame.size +**

**log10(file.size):index.type + chunks:index.type + frame.size:index.type +**

**log10(file.size):chunks:index.type + log10(file.size):frame.size:index.type +**

**chunks:frame.size:index.type | index.type * chunks, data = dat)**

Standardized weighted residuals 2:

Min 1Q Median 3Q Max

-2.8771 -0.7693 -0.2106 0.5468 6.6999

*** LOOK AT THE LINK FUNCTION

* Re-Run model with just 2 way interactions

* present test statistic (z value)t, p value, degrees of freedom

|  | Estimate | Std. Error | z value | Pr(>\|z\|) |
| --- | --- | --- | --- | --- |
| (Intercept) | 1.104521 | 0.100877 | 10.949 | < 2e-16 *** |
| log10(file.size) | -0.015526 | 0.072085 | -0.215 | 0.82946 |
| chunks4 | 0.083034 | 0.130390 | 0.637 | 0.52425 |
| chunks8 | 0.138091 | 0.131426 | 1.051 | 0.29339 |
| chunks12 | -0.058062 | 0.121445 | -0.478 | 0.63259 |
| frame.size10min | -0.070346 | 0.124861 | -0.563 | 0.57317 |
| frame.size5min | 0.031137 | 0.125578 | 0.248 | 0.80417 |
| frame.size2_5min | 0.055264 | 0.125603 | 0.440 | 0.65995 |
| index.typeAudioSet | 1.334952 | 0.128606 | 10.380 | < 2e-16 *** |
| log10(file.size):chunks4 | 0.046597 | 0.080474 | 0.579 | 0.56257 |
| log10(file.size):chunks8 | -0.138090 | 0.080784 | -1.709 | 0.08738 . |
| log10(file.size):chunks12 | -0.160987 | 0.074907 | -2.149 | 0.03162 * |
| log10(file.size):frame.size10min | 0.101558 | 0.079187 | 1.283 | 0.19967 |
| log10(file.size):frame.size5min | 0.038708 | 0.079340 | 0.488 | 0.62564 |
| log10(file.size):frame.size2_5min | 0.007661 | 0.079262 | 0.097 | 0.92300 |
| chunks4:frame.size10min | -0.006499 | 0.139389 | -0.047 | 0.96281 |
| chunks8:frame.size10min | 0.056064 | 0.139326 | 0.402 | 0.68740 |
| chunks12:frame.size10min | 0.061023 | 0.128661 | 0.474 | 0.63529 |
| chunks4:frame.size5min | -0.043339 | 0.139628 | -0.310 | 0.75627 |
| chunks8:frame.size5min | -0.032370 | 0.139174 | -0.233 | 0.81608 |
| chunks12:frame.size5min | 0.061505 | 0.129137 | 0.476 | 0.63388 |
| chunks4:frame.size2_5min | -0.091839 | 0.139014 | -0.661 | 0.50884 |
| chunks8:frame.size2_5min | -0.025140 | 0.139089 | -0.181 | 0.85656 |
| chunks12:frame.size2_5min | 0.082253 | 0.129125 | 0.637 | 0.52412 |
| log10(file.size):index.typeAudioSet | 0.202437 | 0.095711 | 2.115 | 0.03442 * |
| chunks4:index.typeAudioSet | -0.234469 | 0.210918 | -1.112 | 0.26628 |
| chunks8:index.typeAudioSet | 0.060060 | 0.176105 | 0.341 | 0.73307 |
| chunks12:index.typeAudioSet | 0.166156 | 0.178544 | 0.931 | 0.35205 |
| frame.size10min:index.typeAudioSet | 0.002228 | 0.163241 | 0.014 | 0.98911 |
| frame.size5min:index.typeAudioSet | -0.122698 | 0.163322 | -0.751 | 0.45249 |
| frame.size2_5min:index.typeAudioSet | -0.165346 | 0.162560 | -1.017 | 0.30909 |
| log10(file.size):chunks4:index.typeAudioSet | 0.420832 | 0.130630 | 3.222 | 0.00127 ** |
| log10(file.size):chunks8:index.typeAudioSet | 0.177120 | 0.106759 | 1.659 | 0.09710 . |
| log10(file.size):chunks12:index.typeAudioSet | 0.273251 | 0.107516 | 2.541 | 0.01104 * |
| log10(file.size):frame.size10min:index.typeAudioSet | -0.085818 | 0.112621 | -0.762 | 0.44605 |
| log10(file.size):frame.size5min:index.typeAudioSet | -0.027098 | 0.112236 | -0.241 | 0.80921 |
| log10(file.size):frame.size2_5min:index.typeAudioSet | -0.077750 | 0.111174 | -0.699 | 0.48433 |
| chunks4:frame.size10min:index.typeAudioSet | -0.004506 | 0.233281 | -0.019 | 0.98459 |
| chunks8:frame.size10min:index.typeAudioSet | -0.240160 | 0.188273 | -1.276 | 0.20210 |
| chunks12:frame.size10min:index.typeAudioSet | -0.234850 | 0.188868 | -1.243 | 0.21370 |
| chunks4:frame.size5min:index.typeAudioSet | -0.171315 | 0.229595 | -0.746 | 0.45557 |
| chunks8:frame.size5min:index.typeAudioSet | -0.176300 | 0.187565 | -0.940 | 0.34725 |
| chunks12:frame.size5min:index.typeAudioSet | -0.315394 | 0.188434 | -1.674 | 0.09418 . |
| chunks4:frame.size2_5min:index.typeAudioSet | -0.148262 | 0.227108 | -0.653 | 0.51387 |
| chunks8:frame.size2_5min:index.typeAudioSet | -0.227651 | 0.185826 | -1.225 | 0.22055 |
| chunks12:frame.size2_5min:index.typeAudioSet | -0.433908 | 0.186840 | -2.322 | 0.02021 * |

**Phi coefficients (precision model with log link):**

|  | Estimate | Std.Error | Z Value | Pr(>\|z\|) |
| --- | --- | --- | --- | --- |
| (Intercept) | 4.9232 | 0.2351 | 20.944 | < 2e-16 *** |
| index.typeAudioSet | 1.6943 | 0.3330 | 5.088 | 3.61e-07 *** |
| chunks4 | -1.6147 | 0.2623 | -6.155 | 7.50e-10 *** |
| chunks8 | -2.4408 | 0.2487 | -9.815 | < 2e-16 *** |
| chunks12 | -2.6482 | 0.2441 | -10.851 | < 2e-16 *** |
| index.typeAudioSet:chunks4 | -1.7679 | 0.3741 | -4.725 | 2.30e-06 *** |
| index.typeAudioSet:chunks8 | -0.6451 | 0.3532 | -1.826 | 0.0678 . |
| index.typeAudioSet:chunks12 | -1.5081 | 0.3476 | -4.339 | 1.43e-05 *** |

---

Signif. codes: 0 '***' 0.001 '**' 0.01 '*' 0.05 '.' 0.1 ' ' 1

Type of estimator: ML (maximum likelihood)

Log-likelihood: 2395 on 54 Df

Pseudo R-squared: 0.427

Number of iterations: 66 (BFGS) + 2 (Fisher scoring)

**PRECISON**

LINEAR MODEL AOV

|  | df | Sum Sq | Mean Sq | F Value | Pr(>F) |
| --- | --- | --- | --- | --- | --- |
| log10(file.size) | 1 | 0.004 | 0.004 | 0.586 | 0.44408 |
| chunks | 3 | 0.122 | 0.041 | 5.355 | 0.00114 ** |
| frame.size | 3 | 0.004 | 0.001 | 0.197 | 0.89841 |
| index.type | 1 | 14.264 | 14.264 | 1883.934 | < 2e-16 *** |
| log10(file.size):chunks | 3 | 0.040 | 0.013 | 1.741 | 0.15658 |
| log10(file.size):frame.size | 3 | 0.005 | 0.002 | 0.238 | 0.86978 |
| chunks:frame.size | 9 | 0.009 | 0.001 | 0.130 | 0.99892 |
| log10(file.size):index.type | 1 | 0.276 | 0.276 | 36.392 | 1.96e-09 *** |
| chunks:index.type | 3 | 0.075 | 0.025 | 3.323 | 0.01906 * |
| frame.size:index.type | 3 | 0.066 | 0.022 | 2.893 | 0.03416 * |
| log10(file.size):chunks:index.type | 3 | 0.022 | 0.007 | 0.987 | 0.39801 |
| log10(file.size):frame.size:index.type | 3 | 0.017 | 0.006 | 0.748 | 0. 52322 |
| chunks:frame.size:index.type | 9 | 0.007 | 0.001 | 0.101 | 0.99962 |
| Residuals | 1753 | 13.273 | 0.008 |  |  |

Signif. codes: 0 ‘***’ 0.001 ‘**’ 0.01 ‘*’ 0.05 ‘.’ 0.1 ‘ ’ 1

BETAREG AOV

Analysis of Deviance Table (Type II tests)

Response: precision_t

|  | Df | Chisq | Pr(>Chisq) |
| --- | --- | --- | --- |
| log10(file.size) | 1 | 21.9304 | 2.827e-06 *** |
| chunks | 3 | 29.6890 | 1.604e-06 *** |
| frame.size | 3 | 19.7551 | 0.0001908 *** |
| index.type | 1 | 3047.6774 | < 2.2e-16 *** |
| log10(file.size):chunks | 3 | 16.5601 | 0.0008703 *** |
| log10(file.size):frame.size | 3 | 2.8885 | 0.4091298 |
| chunks:frame.size | 9 | 8.2666 | 0.5075198 |
| log10(file.size):index.type | 1 | 70.1343 | < 2.2e-16 *** |
| chunks:index.type | 3 | 10.0553 | 0.0181017 * |
| frame.size:index.type | 3 | 37.7504 | 3.192e-08 *** |
| log10(file.size):chunks:index.type | 3 | 12.2136 | 0.0066863 ** |
| log10(file.size):frame.size:index.type | 3 | 1.2606 | 0.7385054 |
| chunks:frame.size:index.type | 9 | 7.0018 | 0.6369337 |

Signif. codes: 0 ‘***’ 0.001 ‘**’ 0.01 ‘*’ 0.05 ‘.’ 0.1 ‘ ’ 1

Maxima Model Summary:

Call:

betareg(formula = precision_t ~ log10(file.size) + chunks + frame.size + index.type + log10(file.size):chunks +

log10(file.size):frame.size + chunks:frame.size + log10(file.size):index.type + chunks:index.type + frame.size:index.type +

log10(file.size):chunks:index.type + log10(file.size):frame.size:index.type + chunks:frame.size:index.type |

index.type * chunks, data = dat)

Standardized weighted residuals 2:

Min 1Q Median 3Q Max

-3.4204 -0.7584 -0.2320 0.5653 6.9159

Coefficients (mean model with logit link):

|  | Estimate | Std.Error | Z Value | Pr(>\|z\|) |
| --- | --- | --- | --- | --- |
| (Intercept) | 1.181140 | 0.102133 | 11.565 | < 2e-16 *** |
| log10(file.size) | -0.078810 | 0.072539 | -1.086 | 0.27728 |
| chunks4 | 0.128346 | 0.132032 | 0.972 | 0.33101 |
| chunks8 | 0.162282 | 0.132533 | 1.224 | 0.22078 |
| chunks12 | 0.033704 | 0.122106 | 0.276 | 0.78253 |
| frame.size10min | -0.088089 | 0.125963 | -0.699 | 0.48435 |
| frame.size5min | 0.003497 | 0.126647 | 0.028 | 0.97797 |
| frame.size2_5min | 0.035712 | 0.126740 | 0.282 | 0.77812 |
| index.typeAudioSet | 1.258609 | 0.129528 | 9.717 | < 2e-16 *** |
| log10(file.size):chunks4 | 0.048827 | 0.081153 | 0.602 | 0.54740 |
| log10(file.size):chunks8 | -0.095730 | 0.081253 | -1.178 | 0.23873 |
| log10(file.size):chunks12 | -0.126488 | 0.074918 | -1.688 | 0.09134 . |
| log10(file.size):frame.size10min | 0.118991 | 0.079432 | 1.498 | 0.13413 |
| log10(file.size):frame.size5min | 0.069972 | 0.079556 | 0.880 | 0.37912 |
| log10(file.size):frame.size2_5min | 0.039836 | 0.079465 | 0.501 | 0.61616 |
| chunks4:frame.size10min | -0.019950 | 0.140254 | -0.142 | 0.88689 |
| chunks8:frame.size10min | 0.061675 | 0.140020 | 0.440 | 0.65959 |
| chunks12:frame.size10min | 0.042094 | 0.128671 | 0.327 | 0.74356 |
| chunks4:frame.size5min | -0.058843 | 0.140566 | -0.419 | 0.67550 |
| chunks8:frame.size5min | -0.039343 | 0.139823 | -0.281 | 0.77842 |
| chunks12:frame.size5min | 0.026060 | 0.129143 | 0.202 | 0.84008 |
| chunks4:frame.size2_5min | -0.106103 | 0.140104 | -0.757 | 0.44886 |
| chunks8:frame.size2_5min | -0.055055 | 0.139700 | -0.394 | 0.69351 |
| chunks12:frame.size2_5min | 0.020382 | 0.129112 | 0.158 | 0.87456 |
| log10(file.size):index.typeAudioSet | 0.262357 | 0.095836 | 2.738 | 0.00619 ** |
| chunks4:index.typeAudioSet | -0.225830 | 0.209074 | -1.080 | 0.28008 |
| chunks8:index.typeAudioSet | 0.145267 | 0.175036 | 0.830 | 0.40658 |
| chunks12:index.typeAudioSet | 0.225946 | 0.177678 | 1.272 | 0.20349 |
| frame.size10min:index.typeAudioSet | 0.027811 | 0.163868 | 0.170 | 0.86523 |
| frame.size5min:index.typeAudioSet | -0.100711 | 0.163807 | -0.615 | 0.53868 |
| frame.size2_5min:index.typeAudioSet | -0.145433 | 0.163105 | -0.892 | 0.37258 |
| log10(file.size):chunks4:index.typeAudioSet | 0.421647 | 0.129360 | 3.259 | 0.00112 ** |
| log10(file.size):chunks8:index.typeAudioSet | 0.134391 | 0.105921 | 1.269 | 0.20452 |
| log10(file.size):chunks12:index.typeAudioSet | 0.236834 | 0.106627 | 2.221 | 0.02634 * |
| log10(file.size):frame.size10min:index.typeAudioSet | -0.105328 | 0.112208 | -0.939 | 0.34789 |
| log10(file.size):frame.size5min:index.typeAudioSet | -0.049108 | 0.111744 | -0.439 | 0.66032 |
| log10(file.size):frame.size2_5min:index.typeAudioSet | -0.106684 | 0.110651 | -0.964 | 0.33497 |
| chunks4:frame.size10min:index.typeAudioSet | 0.005458 | 0.230916 | 0.024 | 0.98114 |
| chunks8:frame.size10min:index.typeAudioSet | -0.262740 | 0.186811 | -1.406 | 0.15959 |
| chunks12:frame.size10min:index.typeAudioSet | -0.233415 | 0.187695 | -1.244 | 0.21365 |
| chunks4:frame.size5min:index.typeAudioSet | -0.154663 | 0.227259 | -0.681 | 0.49615 |
| chunks8:frame.size5min:index.typeAudioSet | -0.189694 | 0.186022 | -1.020 | 0.30785 |
| chunks12:frame.size5min:index.typeAudioSet | -0.310507 | 0.187116 | -1.659 | 0.09703 . |
| chunks4:frame.size2_5min:index.typeAudioSet | -0.135128 | 0.224834 | -0.601 | 0.54783 |
| chunks8:frame.size2_5min:index.typeAudioSet | -0.224587 | 0.184237 | -1.219 | 0.22284 |
| chunks12:frame.size2_5min:index.typeAudioSet | -0.409874 | 0.185403 | -2.211 | 0.02706 * |

Phi coefficients (precision model with log link):

|  | Estimate | Std.Error | Z Value | Pr(>\|z\|) |
| --- | --- | --- | --- | --- |
| (Intercept) | 4.9166 | 0.2351 | 20.916 | < 2e-16 *** |
| index.typeAudioSet | 1.6912 | 0.3330 | 5.079 | 3.79e-07 *** |
| chunks4 | -1.5953 | 0.2624 | -6.080 | 1.20e-09 *** |
| chunks8 | -2.4061 | 0.2488 | -9.672 | < 2e-16 *** |
| chunks12 | -2.5716 | 0.2442 | -10.532 | < 2e-16 *** |
| index.typeAudioSet:chunks4 - | 1.6599 | 0.3740 | -4.438 | 9.10e-06 *** |
| index.typeAudioSet:chunks8 | -0.4915 | 0.3533 | -1.391 | 0.164 |
| index.typeAudioSet:chunks12 | -1.3908 | 0.3477 | -4.001 | 6.32e-05 *** |

Signif. codes: 0 '***' 0.001 '**' 0.01 '*' 0.05 '.' 0.1 ' ' 1

Type of estimator: ML (maximum likelihood)

Log-likelihood: 2529 on 54 Df

Pseudo R-squared: 0.4346

Number of iterations: 66 (BFGS) + 2 (Fisher scoring)

**RECALL**

LINEAR MODEAL AOV

|  | Df | Sum.Sq | Mean.Sq | F Value | Pr(>F) |
| --- | --- | --- | --- | --- | --- |
| log10(file.size) | 1 | 0.000 | 0.000 | 0.027 | 0.86948 |
| chunks | 3 | 0.241 | 0.080 | 9.581 | 2.83e-06 *** |
| frame.size | 3 | 0.010 | 0.003 | 0.394 | 0.75750 |
| index.type | 1 | 16.000 | 16.000 | 1904.901 | < 2e-16 *** |
| log10(file.size):chunks | 3 | 0.045 | 0.015 | 1.799 | 0.14531 |
| log10(file.size):frame.size | 3 | 0.007 | 0.002 | 0.288 | 0.83382 |
| chunks:frame.size | 9 | 0.016 | 0.002 | 0.214 | 0.99248 |
| log10(file.size):index.type | 1 | 0.300 | 0.300 | 35.664 | 2.83e-09 *** |
| chunks:index.type | 3 | 0.131 | 0.044 | 5.198 | 0.00142 ** |
| frame.size:index.type | 3 | 0.084 | 0.028 | 3.340 | 0.01862 * |
| log10(file.size):chunks:index.type | 3 | 0.041 | 0.014 | 1.625 | 0.18156 |
| log10(file.size):frame.size:index.type | 3 | 0.017 | 0.006 | 0.662 | 0.57556 |
| chunks:frame.size:index.type | 9 | 0.009 | 0.001 | 0.116 | 0.99931 |
| Residuals | 1753 | 14.724 | 0.008 |  |  |

Signif. codes: 0 ‘***’ 0.001 ‘**’ 0.01 ‘*’ 0.05 ‘.’ 0.1 ‘ ’ 1

BETA REG AOV

Analysis of Deviance Table (Type II tests)

|  | Df | Chisq | Pr(>Chisq) |
| --- | --- | --- | --- |
| log10(file.size) | 1 | 21.9304 | 2.827e-06 *** |
| chunks | 3 | 29.6890 | 1.604e-06 *** |
| frame.size | 3 | 19.7551 | 0.0001908 *** |
| index.type | 1 | 3047.6774 | < 2.2e-16 *** |
| log10(file.size):chunks | 3 | 16.5601 | 0.0008703 *** |
| log10(file.size):frame.size | 3 | 2.8885 | 0.4091298 |
| chunks:frame.size | 9 | 8.2666 | 0.5075198 |
| log10(file.size):index.type | 1 | 70.1343 | < 2.2e-16 *** |
| chunks:index.type | 3 | 10.0553 | 0.0181017 * |
| frame.size:index.type | 3 | 37.7504 | 3.192e-08 *** |
| log10(file.size):chunks:index.type | 3 | 12.2136 | 0.0066863 ** |
| log10(file.size):frame.size:index.type | 3 | 1.2606 | 0.7385054 |
| chunks:frame.size:index.type | 9 | 7.0018 | 0.6369337 |

Signif. codes: 0 ‘***’ 0.001 ‘**’ 0.01 ‘*’ 0.05 ‘.’ 0.1 ‘ ’ 1

Maxima Model Summary

Call:

betareg(formula = recall_t ~ log10(file.size) + chunks + frame.size + index.type + log10(file.size):chunks + log10(file.size):frame.size +

chunks:frame.size + log10(file.size):index.type + chunks:index.type + frame.size:index.type + log10(file.size):chunks:index.type +

log10(file.size):frame.size:index.type + chunks:frame.size:index.type | index.type * chunks, data = dat)

Standardized weighted residuals 2:

Min 1Q Median 3Q Max

-2.8994 -0.7530 -0.2073 0.5620 6.7930

Coefficients (mean model with logit link):

|  | Estimate | Std.Error | Z Value | Pr(>\|z\|) |
| --- | --- | --- | --- | --- |
| (Intercept) | 1.098143 | 0.101391 | 10.831 | < 2e-16 *** |
| log10(file.size) | -0.015687 | 0.072412 | -0.217 | 0.82849 |
| chunks4 | 0.090868 | 0.131462 | 0.691 | 0.48943 |
| chunks8 | 0.141348 | 0.131676 | 1.073 | 0.28307 |
| chunks12 | -0.043664 | 0.121815 | -0.358 | 0.72001 |
| frame.size10min | -0.067906 | 0.125422 | -0.541 | 0.58822 |
| frame.size5min | 0.036177 | 0.126179 | 0.287 | 0.77433 |
| frame.size2_5min | 0.061919 | 0.126215 | 0.491 | 0.62372 |
| index.typeAudioSet | 1.330295 | 0.130164 | 10.220 | < 2e-16 *** |
| log10(file.size):chunks4 | 0.041886 | 0.081174 | 0.516 | 0.60586 |
| log10(file.size):chunks8 | -0.136666 | 0.080990 | -1.687 | 0.09152 . |
| log10(file.size):chunks12 | -0.161706 | 0.075030 | -2.155 | 0.03114 * |
| log10(file.size):frame.size10min | 0.102739 | 0.079372 | 1.294 | 0.19553 |
| log10(file.size):frame.size5min | 0.038478 | 0.079556 | 0.484 | 0.62863 |
| log10(file.size):frame.size2_5min | 0.006807 | 0.079479 | 0.086 | 0.93175 |
| chunks4:frame.size10min | -0.006198 | 0.140491 | -0.044 | 0.96481 |
| chunks8:frame.size10min | 0.058158 | 0.139619 | 0.417 | 0.67701 |
| chunks12:frame.size10min | 0.051066 | 0.128978 | 0.396 | 0.69216 |
| chunks4:frame.size5min | -0.038412 | 0.140795 | -0.273 | 0.78499 |
| chunks8:frame.size5min | -0.028125 | 0.139501 | -0.202 | 0.84022 |
| chunks12:frame.size5min | 0.054384 | 0.129486 | 0.420 | 0.67449 |
| chunks4:frame.size2_5min | -0.087356 | 0.140184 | -0.623 | 0.53319 |
| chunks8:frame.size2_5min | -0.022520 | 0.139418 | -0.162 | 0.87168 |
| chunks12:frame.size2_5min | 0.071848 | 0.129468 | 0.555 | 0.57893 |
| log10(file.size):index.typeAudioSet | 0.201887 | 0.096762 | 2.086 | 0.03694 * |
| chunks4:index.typeAudioSet | -0.245409 | 0.212017 | -1.158 | 0.24707 |
| chunks8:index.typeAudioSet | 0.058168 | 0.176588 | 0.329 | 0.74186 |
| chunks12:index.typeAudioSet | 0.163712 | 0.179160 | 0.914 | 0.36083 |
| frame.size10min:index.typeAudioSet | 0.003025 | 0.165081 | 0.018 | 0.98538 |
| frame.size5min:index.typeAudioSet | -0.130519 | 0.165120 | -0.790 | 0.42926 |
| frame.size2_5min:index.typeAudioSet | -0.173535 | 0.164352 | -1.056 | 0.29103 |
| log10(file.size):chunks4:index.typeAudioSet | 0.431582 | 0.131410 | 3.284 | 0.00102 ** |
| log10(file.size):chunks8:index.typeAudioSet | 0.188465 | 0.107162 | 1.759 | 0.07863 . |
| log10(file.size):chunks12:index.typeAudioSet | 0.288612 | 0.107881 | 2.675 | 0.00747 ** |
| log10(file.size):frame.size10min:index.typeAudioSet | -0.088741 | 0.113542 | -0.782 | 0.43447 |
| log10(file.size):frame.size5min:index.typeAudioSet | -0.024313 | 0.113140 | -0.215 | 0.82985 |
| log10(file.size):frame.size2_5min:index.typeAudioSet | -0.075012 | 0.112071 | -0.669 | 0.50329 |
| chunks4:frame.size10min:index.typeAudioSet | -0.003125 | 0.234520 | -0.013 | 0.98937 |
| chunks8:frame.size10min:index.typeAudioSet | -0.242249 | 0.188956 | -1.282 | 0.19983 |
| chunks12:frame.size10min:index.typeAudioSet | -0.230048 | 0.189657 | -1.213 | 0.22514 |
| chunks4:frame.size5min:index.typeAudioSet | -0.173004 | 0.230870 | -0.749 | 0.45364 |
| chunks8:frame.size5min:index.typeAudioSet | -0.181134 | 0.188242 | -0.962 | 0.33593 |
| chunks12:frame.size5min:index.typeAudioSet | -0.316069 | 0.189201 | -1.671 | 0.09481 . |
| chunks4:frame.size2_5min:index.typeAudioSet | -0.151230 | 0.228365 | -0.662 | 0.50783 |
| chunks8:frame.size2_5min:index.typeAudioSet | -0.229031 | 0.186516 | -1.228 | 0.21947 |
| chunks12:frame.size2_5min:index.typeAudioSet | -0.428817 | 0.187602 | -2.286 | 0.02227 * |

Phi coefficients (precision model with log link):

|  | Estimate | Std.Error | Z Value | Pr(>\|z\|) |
| --- | --- | --- | --- | --- |
| (Intercept) | 4.9081 | 0.2350 | 20.881 | < 2e-16 *** |
| index.typeAudioSet | 1.6413 | 0.3330 | 4.929 | 8.26e-07 *** |
| chunks4 | -1.6202 | 0.2623 | -6.176 | 6.56e-10 *** |
| chunks8 | -2.4222 | 0.2487 | -9.740 | < 2e-16 *** |
| chunks12 | -2.6224 | 0.2441 | -10.744 | < 2e-16 *** |
| index.typeAudioSet:chunks4 | -1.7007 | 0.3741 | -4.546 | 5.47e-06 *** |
| index.typeAudioSet:chunks8 - | 0.5817 | 0.3532 | -1.647 | 0.0996 . |
| index.typeAudioSet:chunks12 - | 1.4477 | 0.3476 | -4.165 | 3.12e-05 *** |

---

Signif. codes: 0 '***' 0.001 '**' 0.01 '*' 0.05 '.' 0.1 ' ' 1

Type of estimator: ML (maximum likelihood)

Log-likelihood: 2401 on 54 Df

Pseudo R-squared: 0.4293

Number of iterations: 66 (BFGS) + 2 (Fisher scoring)

9d: Treating Parameters as Continuous:

**ACCURACY**

> max_model <- accuracy_t ~ (log10(file.size) + chunks + frame.size) ^ 2 * index.type

> # Linear Model (as in early analysis)

> mod_lm <- lm(max_model, data= dat)

> summary.aov(mod_lm)

Df Sum Sq Mean Sq F value Pr(>F)

log10(file.size) 1 0.000 0.000 0.000 0.99738

chunks 1 0.194 0.194 23.308 1.50e-06 ***

frame.size 1 0.005 0.005 0.657 0.41767

index.type 1 15.980 15.980 1917.358 < 2e-16 ***

log10(file.size):chunks 1 0.026 0.026 3.091 0.07892 .

log10(file.size):frame.size 1 0.000 0.000 0.036 0.84963

chunks:frame.size 1 0.012 0.012 1.429 0.23213

log10(file.size):index.type 1 0.288 0.288 34.563 4.91e-09 ***

chunks:index.type 1 0.103 0.103 12.356 0.00045 ***

frame.size:index.type 1 0.074 0.074 8.826 0.00301 **

log10(file.size):chunks:index.type 1 0.035 0.035 4.236 0.03972 *

log10(file.size):frame.size:index.type 1 0.003 0.003 0.339 0.56030

chunks:frame.size:index.type 1 0.006 0.006 0.763 0.38257

Residuals 1785 14.876 0.008

---

Signif. codes: 0 ‘***’ 0.001 ‘**’ 0.01 ‘*’ 0.05 ‘.’ 0.1 ‘ ’ 1

**> # Betareg Model**

**> mod_br <- betareg(max_model, data=dat)**

**> # Including Index Type and Chunks into the precision component of the model**

**> mod_br_phi <- update(mod_br, . ~ . | index.type * chunks)**

**> summary(mod_br_phi)**

Call:

betareg(formula = accuracy_t ~ log10(file.size) + chunks + frame.size + index.type + log10(file.size):chunks +

log10(file.size):frame.size + chunks:frame.size + log10(file.size):index.type + chunks:index.type + frame.size:index.type +

log10(file.size):chunks:index.type + log10(file.size):frame.size:index.type + chunks:frame.size:index.type |

index.type * chunks, data = dat)

Standardized weighted residuals 2:

Min 1Q Median 3Q Max

-2.9508 -0.7654 -0.2207 0.4747 6.2269

Coefficients (mean model with logit link):

Estimate Std. Error z value Pr(>|z|)

**(Intercept) 1.147e+00 1.121e-01 10.233 < 2e-16 *****

log10(file.size) 9.695e-02 8.097e-02 1.197 0.23121

chunks 5.030e-03 1.210e-02 0.416 0.67755

frame.size 4.086e-05 1.323e-04 0.309 0.75745

**index.typeAudioSet 9.345e-01 1.762e-01 5.302 1.14e-07 *****

**log10(file.size):chunks -1.999e-02 8.032e-03 -2.489 0.01280 ***

log10(file.size):frame.size -2.056e-05 7.761e-05 -0.265 0.79114

chunks:frame.size -1.010e-05 1.222e-05 -0.827 0.40822

**log10(file.size):index.typeAudioSet 3.420e-01 1.315e-01 2.601 0.00929 ****

chunks:index.typeAudioSet -1.252e-03 1.908e-02 -0.066 0.94765

frame.size:index.typeAudioSet 1.300e-04 2.144e-04 0.607 0.54417

log10(file.size):chunks:index.typeAudioSet 4.399e-04 1.299e-02 0.034 0.97298

log10(file.size):frame.size:index.typeAudioSet 1.370e-04 1.269e-04 1.079 0.28045

chunks:frame.size:index.typeAudioSet 2.517e-05 2.027e-05 1.242 0.21435

Phi coefficients (precision model with log link):

Estimate Std. Error z value Pr(>|z|)

**(Intercept) 3.686964 0.129049 28.570 <2e-16 *****

**index.typeAudioSet 0.384924 0.185972 2.070 0.0385 ***

**chunks -0.125049 0.013364 -9.357 <2e-16 *****

index.typeAudioSet:chunks -0.001346 0.019434 -0.069 0.9448

---

Signif. codes: 0 '***' 0.001 '**' 0.01 '*' 0.05 '.' 0.1 ' ' 1

Type of estimator: ML (maximum likelihood)

Log-likelihood: 2301 on 18 Df

Pseudo R-squared: 0.4209

Number of iterations: 30 (BFGS) + 3 (Fisher scoring)

> AIC(mod_lm, mod_br, mod_br_phi) # shows this model is best

df AIC

mod_lm 15 -3491.238

mod_br 15 -4401.508

mod_br_phi 18 -4565.338

> aov_table <- Anova(mod_br_phi)

> aov_table

Analysis of Deviance Table (Type II tests)

Response: accuracy_t

Df Chisq Pr(>Chisq)

log10(file.size) 1 14.6258 0.0001311 ***

chunks 1 17.2050 3.355e-05 ***

frame.size 1 8.6459 0.0032780 **

index.type 1 1912.4822 < 2.2e-16 ***

log10(file.size):chunks 1 9.8664 0.0016832 **

log10(file.size):frame.size 1 0.2492 0.6176605

chunks:frame.size 1 0.0097 0.9213576

log10(file.size):index.type 1 69.2583 < 2.2e-16 ***

chunks:index.type 1 2.0376 0.1534518

frame.size:index.type 1 34.8975 3.475e-09 ***

log10(file.size):chunks:index.type 1 0.0011 0.9729774

log10(file.size):frame.size:index.type 1 1.1649 0.2804523

chunks:frame.size:index.type 1 1.5418 0.2143490

---

Signif. codes: 0 ‘***’ 0.001 ‘**’ 0.01 ‘*’ 0.05 ‘.’ 0.1 ‘ ’ 1

**PRECISION**

> max_model <- precision_t ~ (log10(file.size) + chunks + frame.size) ^ 2 * index.type

> # Linear Model (as in early analysis)

> mod_lm <- lm(max_model, data= dat)

> summary.aov(mod_lm)

Df Sum Sq Mean Sq F value Pr(>F)

log10(file.size) 1 0.004 0.004 0.592 0.44176

chunks 1 0.074 0.074 9.931 0.00165 **

frame.size 1 0.002 0.002 0.207 0.64929

index.type 1 14.264 14.264 1903.190 < 2e-16 ***

log10(file.size):chunks 1 0.023 0.023 3.072 0.07980 .

log10(file.size):frame.size 1 0.000 0.000 0.042 0.83792

chunks:frame.size 1 0.006 0.006 0.750 0.38654

log10(file.size):index.type 1 0.276 0.276 36.768 1.62e-09 ***

chunks:index.type 1 0.061 0.061 8.203 0.00423 **

frame.size:index.type 1 0.064 0.064 8.533 0.00353 **

log10(file.size):chunks:index.type 1 0.021 0.021 2.741 0.09800 .

log10(file.size):frame.size:index.type 1 0.007 0.007 0.939 0.33259

chunks:frame.size:index.type 1 0.005 0.005 0.649 0.42042

Residuals 1785 13.378 0.007

---

Signif. codes: 0 ‘***’ 0.001 ‘**’ 0.01 ‘*’ 0.05 ‘.’ 0.1 ‘ ’ 1

**> # Betareg Model**

**> mod_br <- betareg(max_model, data=dat)**

**> # Including Index Type and Chunks into the precision component of the model**

**> mod_br_phi <- update(mod_br, . ~ . | index.type * chunks)**

**> summary(mod_br_phi)**

Call:

betareg(formula = precision_t ~ log10(file.size) + chunks + frame.size + index.type + log10(file.size):chunks +

log10(file.size):frame.size + chunks:frame.size + log10(file.size):index.type + chunks:index.type + frame.size:index.type +

log10(file.size):chunks:index.type + log10(file.size):frame.size:index.type + chunks:frame.size:index.type |

index.type * chunks, data = dat)

Standardized weighted residuals 2:

Min 1Q Median 3Q Max

-3.2818 -0.7450 -0.2431 0.4942 6.4455

Coefficients (mean model with logit link):

Estimate Std. Error z value Pr(>|z|)

**(Intercept) 1.199e+00 1.134e-01 10.576 < 2e-16 *****

log10(file.size) 7.168e-02 8.170e-02 0.877 0.38030

chunks 6.611e-03 1.217e-02 0.543 0.58714

frame.size 6.663e-05 1.337e-04 0.498 0.61830

**index.typeAudioSet 8.978e-01 1.742e-01 5.155 2.54e-07 *****

**log10(file.size):chunks -1.622e-02 8.077e-03 -2.008 0.04468 ***

log10(file.size):frame.size -5.560e-05 7.791e-05 -0.714 0.47547

chunks:frame.size -6.978e-06 1.227e-05 -0.569 0.56964

**log10(file.size):index.typeAudioSet 3.686e-01 1.298e-01 2.839 0.00452 ****

chunks:index.typeAudioSet 5.268e-03 1.880e-02 0.280 0.77928

frame.size:index.typeAudioSet 9.215e-05 2.118e-04 0.435 0.66345

log10(file.size):chunks:index.typeAudioSet -3.549e-03 1.280e-02 -0.277 0.78164

log10(file.size):frame.size:index.typeAudioSet 1.709e-04 1.253e-04 1.364 0.17246

chunks:frame.size:index.typeAudioSet 2.604e-05 1.999e-05 1.302 0.19276

Phi coefficients (precision model with log link):

Estimate Std. Error z value Pr(>|z|)

(Intercept) 3.667224 0.129159 28.393 <2e-16 ***

index.typeAudioSet 0.477444 0.185956 2.568 0.0102 *

chunks -0.118561 0.013389 -8.855 <2e-16 ***

index.typeAudioSet:chunks 0.002017 0.019442 0.104 0.9174

---

Signif. codes: 0 '***' 0.001 '**' 0.01 '*' 0.05 '.' 0.1 ' ' 1

Type of estimator: ML (maximum likelihood)

Log-likelihood: 2431 on 18 Df

Pseudo R-squared: 0.4288

Number of iterations: 30 (BFGS) + 2 (Fisher scoring)

> AIC(mod_lm, mod_br, mod_br_phi) # shows this model is best

df AIC

mod_lm 15 -3682.240

mod_br 15 -4654.656

mod_br_phi 18 -4825.003

> aov_table <- Anova(mod_br_phi)

> aov_table

Analysis of Deviance Table (Type II tests)

Response: precision_t

Df Chisq Pr(>Chisq)

log10(file.size) 1 13.3449 0.0002591 ***

chunks 1 4.4200 0.0355203 *

frame.size 1 12.0242 0.0005251 ***

index.type 1 2014.0679 < 2.2e-16 ***

log10(file.size):chunks 1 7.9126 0.0049092 **

log10(file.size):frame.size 1 0.0297 0.8631529

chunks:frame.size 1 0.0855 0.7699322

log10(file.size):index.type 1 76.1929 < 2.2e-16 ***

chunks:index.type 1 3.2384 0.0719289 .

frame.size:index.type 1 36.4003 1.607e-09 ***

log10(file.size):chunks:index.type 1 0.0768 0.7816436

log10(file.size):frame.size:index.type 1 1.8614 0.1724631

chunks:frame.size:index.type 1 1.6964 0.1927592

---

Signif. codes: 0 ‘***’ 0.001 ‘**’ 0.01 ‘*’ 0.05 ‘.’ 0.1 ‘ ’ 1

**RECALL**

> max_model <- recall_t ~ (log10(file.size) + chunks + frame.size) ^ 2 * index.type

> # Linear Model (as in early analysis)

> mod_lm <- lm(max_model, data= dat)

> summary.aov(mod_lm)

Df Sum Sq Mean Sq F value Pr(>F)

log10(file.size) 1 0.000 0.000 0.027 0.868874

chunks 1 0.189 0.189 22.719 2.03e-06 ***

frame.size 1 0.008 0.008 1.008 0.315415

index.type 1 15.999 15.999 1922.764 < 2e-16 ***

log10(file.size):chunks 1 0.023 0.023 2.819 0.093339 .

log10(file.size):frame.size 1 0.000 0.000 0.024 0.876837

chunks:frame.size 1 0.012 0.012 1.445 0.229471

log10(file.size):index.type 1 0.300 0.300 36.002 2.38e-09 ***

chunks:index.type 1 0.112 0.112 13.489 0.000247 ***

frame.size:index.type 1 0.082 0.082 9.880 0.001699 **

log10(file.size):chunks:index.type 1 0.037 0.037 4.410 0.035864 *

log10(file.size):frame.size:index.type 1 0.003 0.003 0.312 0.576262

chunks:frame.size:index.type 1 0.006 0.006 0.765 0.381986

Residuals 1785 14.853 0.008

---

Signif. codes: 0 ‘***’ 0.001 ‘**’ 0.01 ‘*’ 0.05 ‘.’ 0.1 ‘ ’ 1

**> # Betareg Model**

**> mod_br <- betareg(max_model, data=dat)**

**> # Including Index Type and Chunks into the precision component of the model**

**> mod_br_phi <- update(mod_br, . ~ . | index.type * chunks)**

**> summary(mod_br_phi)**

Call:

betareg(formula = recall_t ~ log10(file.size) + chunks + frame.size + index.type + log10(file.size):chunks + log10(file.size):frame.size +

chunks:frame.size + log10(file.size):index.type + chunks:index.type + frame.size:index.type + log10(file.size):chunks:index.type +

log10(file.size):frame.size:index.type + chunks:frame.size:index.type | index.type * chunks, data = dat)

Standardized weighted residuals 2:

Min 1Q Median 3Q Max

-2.9469 -0.7504 -0.2228 0.4802 6.2298

Coefficients (mean model with logit link):

Estimate Std. Error z value Pr(>|z|)

**(Intercept) 1.158e+00 1.128e-01 10.262 < 2e-16 *****

log10(file.size) 9.237e-02 8.146e-02 1.134 0.25681

chunks 4.649e-03 1.216e-02 0.382 0.70212

frame.size 3.086e-05 1.330e-04 0.232 0.81649

**index.typeAudioSet 9.083e-01 1.766e-01 5.142 2.71e-07 *****

**log10(file.size):chunks -1.964e-02 8.068e-03 -2.435 0.01491 ***

log10(file.size):frame.size -1.806e-05 7.780e-05 -0.232 0.81648

chunks:frame.size -1.033e-05 1.226e-05 -0.842 0.39970

**log10(file.size):index.typeAudioSet 3.512e-01 1.318e-01 2.665 0.00769 ****

chunks:index.typeAudioSet -5.484e-04 1.908e-02 -0.029 0.97708

frame.size:index.typeAudioSet 1.406e-04 2.147e-04 0.655 0.51246

log10(file.size):chunks:index.typeAudioSet 1.253e-03 1.300e-02 0.096 0.92325

log10(file.size):frame.size:index.typeAudioSet 1.291e-04 1.269e-04 1.018 0.30884

chunks:frame.size:index.typeAudioSet 2.624e-05 2.029e-05 1.293 0.19587

Phi coefficients (precision model with log link):

Estimate Std. Error z value Pr(>|z|)

(Intercept) 3.6677091 0.1290430 28.422 <2e-16 ***

index.typeAudioSet 0.3899579 0.1859748 2.097 0.036 *

chunks -0.1232328 0.0133649 -9.221 <2e-16 ***

index.typeAudioSet:chunks -0.0003653 0.0194351 -0.019 0.985

---

Signif. codes: 0 '***' 0.001 '**' 0.01 '*' 0.05 '.' 0.1 ' ' 1

Type of estimator: ML (maximum likelihood)

Log-likelihood: 2305 on 18 Df

Pseudo R-squared: 0.4235

Number of iterations: 30 (BFGS) + 2 (Fisher scoring)

> AIC(mod_lm, mod_br, mod_br_phi) # shows this model is best

df AIC

mod_lm 15 -3494.087

mod_br 15 -4412.078

mod_br_phi 18 -4573.955

> aov_table <- Anova(mod_br_phi)

> aov_table

Analysis of Deviance Table (Type II tests)

Response: recall_t

Df Chisq Pr(>Chisq)

log10(file.size) 1 16.0094 6.303e-05 ***

chunks 1 15.9911 6.364e-05 ***

frame.size 1 7.9690 0.004758 **

index.type 1 1913.3500 < 2.2e-16 ***

log10(file.size):chunks 1 9.1696 0.002461 **

log10(file.size):frame.size 1 0.2459 0.619967

chunks:frame.size 1 0.0057 0.939644

log10(file.size):index.type 1 73.0189 < 2.2e-16 ***

chunks:index.type 1 2.7647 0.096362 .

frame.size:index.type 1 36.5939 1.455e-09 ***

log10(file.size):chunks:index.type 1 0.0093 0.923248

log10(file.size):frame.size:index.type 1 1.0356 0.308839

chunks:frame.size:index.type 1 1.6729 0.195866

---

Signif. codes: 0 ‘***’ 0.001 ‘**’ 0.01 ‘*’ 0.05 ‘.’ 0.1 ‘ ’ 1

**10 – References**

Boelman, N. T., Asner, G. P., Hart, P. J., & Martin, R. E. (2007). Multi-trophic invasion resistance in Hawaii: Bioacoustics, field surveys, and airborne remote sensing. *Ecological Applications*, *17*(8), 2137–2144. https://doi.org/10.1890/07-0004.1

Bradfer‐Lawrence, T., Gardner, N., Bunnefeld, L., Bunnefeld, N., Willis, S. G., & Dent, D. H. (2019). Guidelines for the use of acoustic indices in environmental research. *Methods in Ecology and Evolution*, *10*(10), 1796–1807. https://doi.org/10.1111/2041-210x.13254

Kasten, E. P., Gage, S. H., Fox, J., & Joo, W. (2012). The remote environmental assessment laboratory’s acoustic library: An archive for studying soundscape ecology. *Ecological Informatics*, *12*, 50–67. https://doi.org/10.1016/j.ecoinf.2012.08.001

Pieretti, N., Farina, A., & Morri, D. (2011). A new methodology to infer the singing activity of an avian community: The Acoustic Complexity Index (ACI). *Ecological Indicators*, *11*(3), 868–873. https://doi.org/10.1016/j.ecolind.2010.11.005

Sueur, J., Farina, A., Gasc, A., Pieretti, N., & Pavoine, S. (2014). Acoustic indices for biodiversity assessment and landscape investigation. *Acta Acustica United with Acustica*, *100*(4), 772–781. https://doi.org/10.3813/AAA.918757

Sueur, J., Pavoine, S., Hamerlynck, O., & Duvail, S. (2008). Rapid acoustic survey for biodiversity appraisal. *PLoS ONE*, *3*(12). https://doi.org/10.1371/journal.pone.0004065

Villanueva-Rivera, L. J., Pijanowski, B. C., Doucette, J., & Pekin, B. (2011). A primer of acoustic analysis for landscape ecologists. *Landscape Ecology*, *26*(9), 1233–1246. https://doi.org/10.1007/s10980-011-9636-9
